# Unique *Leishmania mexicana* clones secrete populations of extracellular vesicles with unique protein profile and variable infectious capability

**DOI:** 10.3389/fcimb.2024.1443262

**Published:** 2024-12-05

**Authors:** George Dong, Noélie Douanne, Christopher Fernandez-Prada, Martin Olivier

**Affiliations:** ^1^ Infectious Diseases and Immunity in Global Health Program, Research Institute of McGill University Health Centre, Montréal, QC, Canada; ^2^ Department of Pathology and Microbiology, Faculty of Veterinary Medicine, Université de Montréal, Saint-Hyacinthe, QC, Canada; ^3^ Department of Microbiology and Immunology, McGill University, Montréal, QC, Canada

**Keywords:** *Leismania mexicana*, extracellular vesicles, exosomes, clonal cultures, proteomics, infectious disease

## Abstract

The study of extracellular vesicles has become an incredibly important field of study, but the inherent heterogeneity of these vesicles continues to make their study challenging. The genetic variability and well-documented protocols for the growth and vesicle isolation from *Leishmania* parasites provide a unique opportunity to compare the heterogeneity of different populations secreted by *Leishmania* clones. *Leishmania mexicana* was cultured on solid SDM agar plates and 8 clonal colonies were selected. The EVs collected from the liquid cultures of these 8 clones were assessed by NTA, TEM, and proteomic analysis. We found that all 8 clonal *L. mexicana* cultures were visually indistinguishable from each other and had similar growth rate, and these physical similarities extended to their EVs. However, proteomic analysis reveals that the EVs collected have unique protein profiles compared to each other and EVs isolated from a heterogeneous liquid culture of *L. mexicana*. We selected 3 clonal EVs for further mouse infection experiments and found that EVs from CL7 *L. mexicana* consistently caused reduced footpad swelling in C57BL6 mice footpads compared to EVs from CL1, CL8, and heterogenous *L. mexicana*. This trend was not observed when infecting Balb/C mice and C57BL6 with the parasites alone, with only CL1 *L. mexicana* causing significantly increased infection in Balb/c mice. Our results together show that EVs isolated from different clonal colonies of *L. mexicana* have distinct differences in protein cargo which can lead to varying outcomes on *Leishmania* infection. Further evaluation will be needed to determine the underlying mechanisms behind this and verify that differences observed in infectivity are directly caused by variations between our *L. mexicana* clones, especially genetic sequencing and immunoblotting to validate our results.

## Introduction

Extracellular vesicles (EVs) are important vehicles for cell-cell communication and are constitutively secreted by both bacteria and eukaryotes (van Niel et al., 2018). They carry many biologically active macromolecules, ranging from nucleic acids to proteins, from their cell of origin and deliver these to a recipient cell to induce the desired physiological change (Thery et al., 2006). EVs have been of great interest due to their importance in intercellular communication, especially in the context of immune and tumor cell research and have recently been established to play an important role in pathogen-host interaction (Dong et al., 2019). Understanding the mechanisms of EV biogenesis and release/uptake have already been proven to be key to understanding human disease progression and diagnosis, but the study of EVs in pathogens and parasites is still a developing field of research.

*Leishmania* spp. are protozoan parasites that are the causative agents of the neglected tropical disease Leishmaniasis (Mann et al., 2021). These parasites specifically infect the macrophages of mammalian hosts and have evolved several mechanisms to evade the host immune response involving a variety of immune-modulating virulence factors (Olivier et al., 2012). Our lab has extensively studied these immunomodulatory virulence factors, especially GP63, and our research along with that of others in the field has revealed the important role of *Leishmania* EVs as vehicles for the delivery of *Leishmania* virulence factors to target mammalian host cells. *Leishmania* EVs are constitutively secreted and can also be induced by 37C temperature shock (Silverman et al., 2010a; Silverman et al., 2010b; Hassani et al., 2011). These EVs are loaded with common virulence factors like GP63 and EF1a and have profound immunomodulatory effects on mammalian macrophages. *Leishmania* EVs are also constitutively secreted within the midgut of its Sandfly vector and are therefore present during initial infection (Atayde et al., 2015). This co-inoculation causes heightened infection severity.

There are many distinct classes of EVs, including ectosomes, microvesicles, apoptotic bodies, exosomes, exomeres, and more (Colombo et al., 2014). Most recent EV research has focused on exosomes, a class of EVs ranging from 30 to 140nm in diameter. Exosomes originate from the endocytic pathway and first form as intraluminal vesicles within multivesicular bodies (MVB). Eventually, these vesicles are released once the MVB fuses with the plasma membrane. It was this distinct biogenesis pathway with directed cargo sorting that made exosomes particularly attractive to study as the main vehicles for intercellular communication. However, it has been shown that exosomes cannot be defined by consistent biomarkers or a conveniently distinct cellular origin and have instead begun to be classified based on their small size (sEVs) (Ramirez et al., 2018). This heterogeneity of EVs extends further to the subpopulations of EVs that can be secreted by not only different cell types but even individual cells within a single population (Willms et al., 2018).

The study of EV heterogeneity at the single cell level is a relatively recent development and often involve the use of expensive and/or custom-made microfluidic systems (Bordanaba-Florit et al., 2021). However, *Leishmania* spp. present an alternative novel method of shedding light on the variance of EVs. *Leishmania* can be grown in liquid cultures and can also be plated onto solid media. When plated, *Leishmania* forms clonal colonies like many bacteria cultures (Pacheco RS et al., 1990). By selecting clonal colonies of *Leishmania*, genetically identical liquid cultures of *Leishmania* can be expanded and EVs can be collected from these cultures. Following this methodology, 8 different clones of *Leishmania mexicana*, a species with great genetic heterogeneity (Berzunza-Cruz et al., 2000), were selected and the EVs released by these clones were collected using differential ultracentrifugation and compared based on their characteristics and effects on *Leishmania* infection. Sample enrichment was omitted in order to assess the entire population of clonal EVs rather than a single type of EVs alone.

## Methods

### Parasite culture and plating

*L. mexicana* m379 was cultured in 26C liquid Schneider’s Drosophila Medium (Gibco-BRL, Grand Island, NY) supplemented with 10% heat-inactivated standard fetal bovine serum (FBS) (Sigma-Aldrich), 2 mM L-glutamine, 100 U/ml penicillin, and 100 µl/ml streptomycin (complete Schneider’s). To make solid SDM agar, un-supplemented SDM was mixed with 2.5% agar solution and heat inactivated FBS in a ratio of 5:4:1, then this mixture was heated to 56C and poured into 100mm petri dishes to a thickness of 5mm and cooled. 2*10^6 log phase (3-4 day old liquid culture) *L. mexicana* promastigotes were then spread onto the SDM-agar plates and incubated at 26C for 10 days. Individual colonies were then carefully picked under magnification and cultured in liquid SDM. The resulting liquid cultures were then frozen after 3-4 days. Subsequent culturing of frozen *L. mexicana* clones was limited to a maximum of 2 passages.

### EV purification

Purification of *Leishmania* EVs was performed according to standard methods for mammalian cells (Thery et al., 2006; Hassani et al., 2011). *L. mexicana* stationary phase promastigotes (day 6-8) were washed twice with PBS to remove SDM media and especially FBS, resuspended in vesicle-free RPMI 1640 medium (Life Technologies, Rockville, MD) without FBS and phenol red at a concentration of 2-4*10^8 promastigotes/ml, and incubated for 4 hours at 37°C for the release of EVs in the culture medium. At the end of a 4-hour incubation, the sample was centrifuged at 4000 RPM to remove parasites and large debris and the supernatant was filtered through a 0.45 µm syringe filter, followed by a 0.20 µm syringe filter. Next, EVs were pelleted by 2 rounds of 1-hour centrifugation at 100 000 g at 4C and resuspended in exosome buffer (137 mM NaCl, 20 mM Hepes, pH 7.5). Resulting EV samples were aliquoted and frozen at -80C, with each aliquot being thawed no more than twice. EV protein content was determined using Thermo Fisher MicroBCA kit according to manufacturer instructions.

### Transmission electron microscopy

*In vitro* purified *L. mexicana* clone EVs were coated directly onto carbon-coated copper grids by dropping 5 ul on to the grid and allowing to dry for 2 minutes. The grids were then dried using the edge of a Kimwipe and fixed with 1% glutaraldehyde in 0.1 M sodium cacodylate buffer for 1 minute, washed 3 times in miliQ water, and stained with 1% uranyl acetate for 1 minute. After drying, grids were visualized in the FEI Tecnai G2 Spirit Twin 120 kV Cryo-TEM and images were taken with the Gatan Ultrascan 4000 4k x 4k CCD Camera System Model 895. Microscopy and sample preparation was performed at the Facility for Electron Microscopy Research at McGill University.

### Nanoparticle tracking analysis

EV samples were initially diluted by 1:100 in exosome buffer and NTA was performed using the NanoSight NS500 (Malvern Panalytical). Samples were loaded into the machine at a minimum initial volume of 500 uL. Focus and camera level were then adjusted based on the first sample before standard script was run (camera level was unchanged for subsequent samples). Three 30 second videos were taken using the standard camera and analysis was performed using the NTA software to determine particle quantity and size as well as to perform statistical analysis.

### LC/MS-MS

LC-MS/MS was performed as previously described at the Institute de Recherches Cliniques de Montréal (IRCM, University of Montreal, Canada) (Olivier et al., 2012; Atayde et al., 2015; Douanne et al., 2020). EVs were diluted in ultrapure water and protein content was determined using the microBCA protein assay kit (Thermo Scientific) to account for the high dilution ratio (1:20). Protein concentration was determined using an Infinite 200 Pro spectrophotometer (Tecan) and interpolation with a BSA standard curve. EV protein was then precipitated with 15% trichloroacetic acid (TCA)/acetone and processed for LC-MS/MS analysis, utilizing 3ug of protein from each sample of clonal EVs and Ht clonal EVs. After precipitation, in solution digestion was performed by the addition of trypsin at a ratio of 1:25 protease: protein. After an overnight incubation at 37°C, the reactions were quenched by the addition of formic acid to a final concentration of 0.2% and cleaned with C18 Zip Tip pipette tips (Millipore, Billerica, MA), before mass spectrometry analysis. Extracted peptides were injected onto a Zorbax Extended-C18 desalting column (Agilent, Santa Clara, CA) and subsequent chromatography was performed using the Easy-LLC II system (Proxeon Biosystems, Odense, Denmark) and coupled to the LTQ Orbitrap Velos (ThermoFisher Scientific, Bremen, Germany) equipped with a Proxeon nanoelectrospray ion source to perform mass spectrometry.

### Proteomic analysis

Individual sample tandem mass spectrometry spectra were peak listed using the Distiller version 2.1.0.0 software (www.matrixscience.com/distiller) with the following parameters: minimum mass set to 500 Da, maximum mass set to 6,000 Da, no grouping of MS/MS spectra, precursor charge set to auto, minimum number of fragment ions set to 5, and peak picking parameters set at 1 for signal noise ratio and at 0.3 for correlation threshold. The peak-listed data was then searched against the NCBI database with the Mascot software version 2.3.02 (Matrix Science, London, UK). Mascot was set up to search the *L. mexicana* database (txid5665, 17 265 proteins) with a fragment ion mass tolerance of 0.50 Da and a parent ion tolerance of 1.5 Da. Carbamidomethyl was specified in both search engines as a fixed modification. Oxidation of methionine residues was specified in Mascot as a variable modification. Scaffold software version 4.0.6.1 (Proteome Software Inc., Portland, OR) was used to validate MS/MS peptide and protein identifications. Identification of peptides was accepted if it could be established at greater than 95.0% probability. Identification of proteins was accepted if it could be established at greater than 95.0% probability and contained at least 2 identified peptides. Proteins that contained similar peptides and could not be differentiated based on MS/MS analysis alone were grouped to satisfy the principles of parsimony. The final number of peptides per protein was represented by the average of the biological replicas after normalization to the total number of peptides. Unidentified proteins were removed from analysis. Unique and shared proteins were compared between different *L. mexicana* clones using UpSet (Lex et al., 2014). Gene ontology (GO) annotations were attributed using Panther by converting *L. mexicana* uniprot accession IDs to *Leishmania major* to functionally classify each protein (Mi et al., 2019). *L. mexicana* interacting EV proteins were mapped using STRING, searching against *L. major* as the STRING database lacks *L. mexicana* (Szklarczyk et al., 2019). The full STRING network was used with interactions determined based on evidence and a medium minimum interaction score (0.400). Clusters were generated using k-means clustering with 4 clusters and interactions were determined based on textmining, experiments, databases, co-expression, neighborhood, gene fusion, and co-occurence.

### Mice infections

All experiments with mice were carried out in pathogen-free housing and in accordance with the regulations of the Canadian Council of Animal Care, approved by the McGill University Animal Care Committee. Female C57BL6 mice (6 to 8 weeks old) were infected in the right hind footpad with 5*10^6^ stationary phase *L. mexicana* Ht promastigotes and 5 µg of *in vitro* purified *L. mexicana* Ht, CL1, 7, and 8 EVs. Female C57BL6 mice were also infected in the right hind footpad with 5*10^6^ *L. mexicana* Ht, CL1, 7, and 8 promastigotes without vesicle co-inoculation. This infection was also performed using male BALB/c mice (6 to 8 weeks old). Disease progression was monitored by measuring footpad swelling weekly with a metric caliper, up to 10 weeks post-infection. At the end of each experiment, footpads were imaged. Statistical analysis was performed using Graphpad Prism 8.

## Results

### *L. mexicana* clone EVs have similar size and morphology

**Figure 1:**
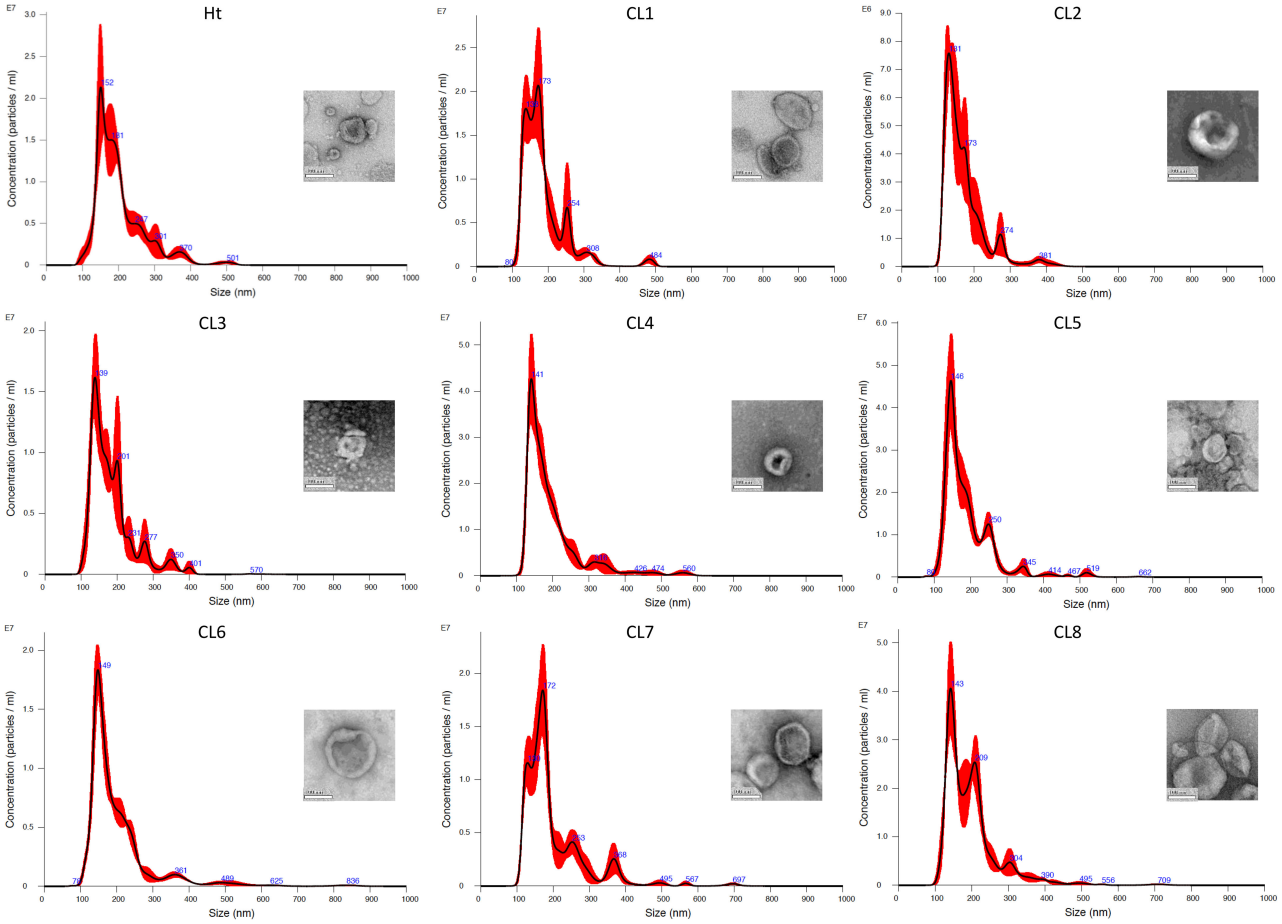
Particle diameter distribution of EVs isolated from each *L. mexicana* clone and Ht graphed by Malvern Panalytical NTA software. Example TEM images of the EVs are also provided, each having been taken at 30 000x magnification, with the scale bar representing 100nm.

**Figure 2:**
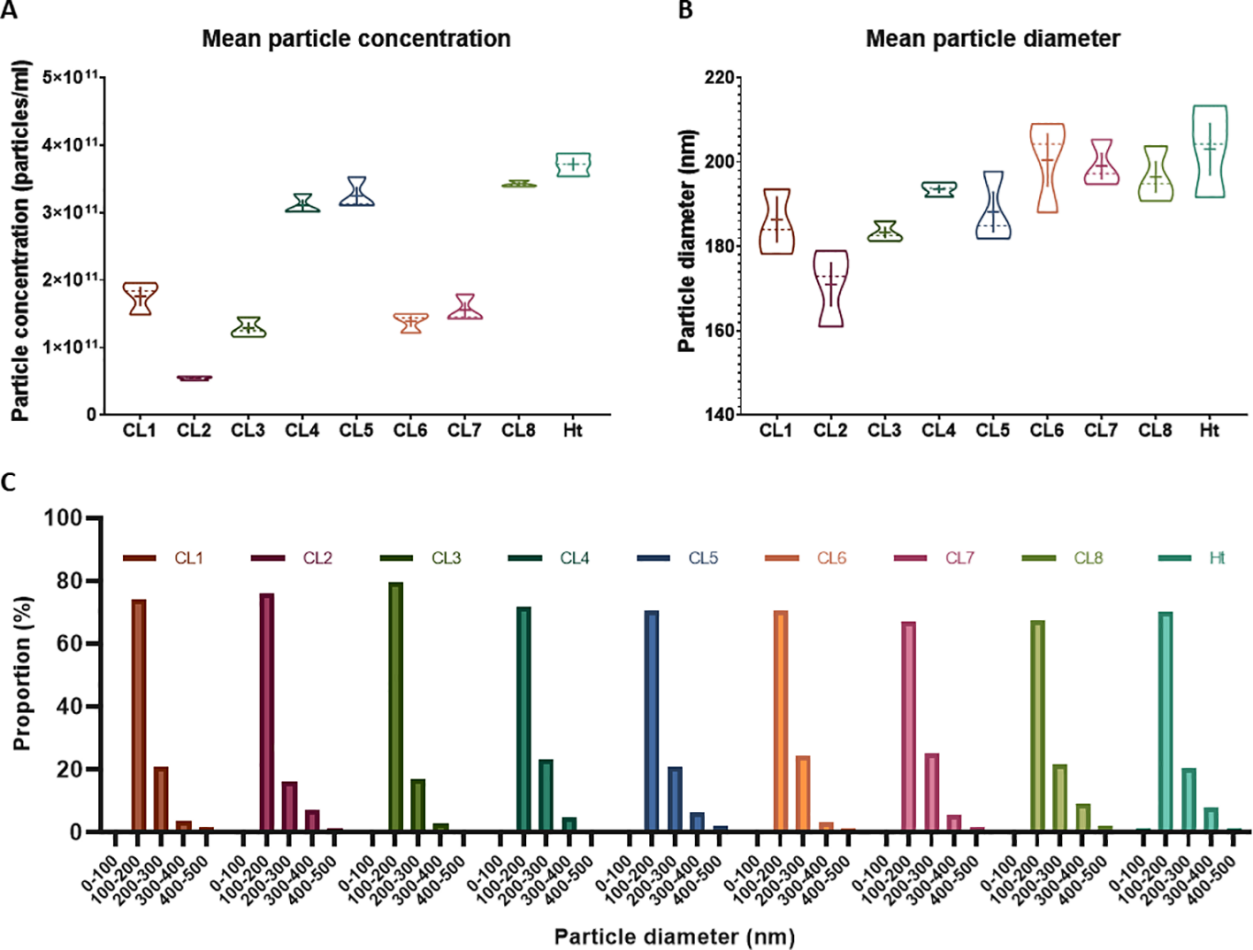
Mean particle concentration and size were compared between the *L. mexicana* clones and Ht *L. mexicana* using violin plots **(A, B)**. The solid line represents the mean while the dotted line represents the median. Proportional size distribution of EVs isolated from each *L. mexicana* clone and Ht was also graphed and to compare overall variations between the EV populations **(C)**. Particle concentration is represented as proportional to the total EV concentration of each sample.

Without further enrichment, the EV samples collected for this study are comprised of multiple types of EVs, mostly a mixture of exosomes and microvesicles after filtration. NTA was performed on these EVs isolated from 8 different *L. mexicana* cultures grown from clonal colonies, as well as an Ht *L. mexicana* culture, using the NS500. The average concentration, mean particle diameter, and mode particle diameter were recorded (**Table 1**) and the particle size distribution of each EV population was graphed using the Nanosight NTA software (**Figure 1**). Particle concentration of each *L. mexicana* clone EV and the Ht EVs were compared, with CL2 having the lowest particle concentration and Ht having the highest (**Figure 2A**). However, no significant difference in particle concentration was found between the clones or Ht. Mean particle size (**Figure 2B**) and proportional size distribution (**Figure 2C**) were also compared, with no significant difference found in either comparison, though CL2 EVs had much lower mean and median particle size. TEM imaging was also performed to confirm successful EV isolation (**Figure 1**). Well defined EVs were identified in all samples, demonstrating a circular shape with the signature cupped form due to sample drying. The EVs were visually indistinguishable between samples.

**Table 1:** Mean concentration, mean particle diameter, and mode particle diameter of EVs isolated from *L. mexicana* Clones and Ht. Stats were determined by the NS500 and NTA software from Malvern Panalytical from 3 separate videos for each sample.

*L. mexicana* culture	Mean particle concentration (particles/ml)	Mean particle diameter (nm)	Mode particle diameter (nm)
CL1	1.76E11	185	159
CL2	5.47E10	171	149
CL3	1.29E11	183	137
CL4	3.11E11	194	148
CL5	3.25E11	188	153
CL6	1.39E11	201	148
CL7	1.56E11	199	168
CL8	3.43E11	197	156
Ht	3.72E11	203	151

### EV protein content varies between different *L. mexicana* clones

**Figure 3:**
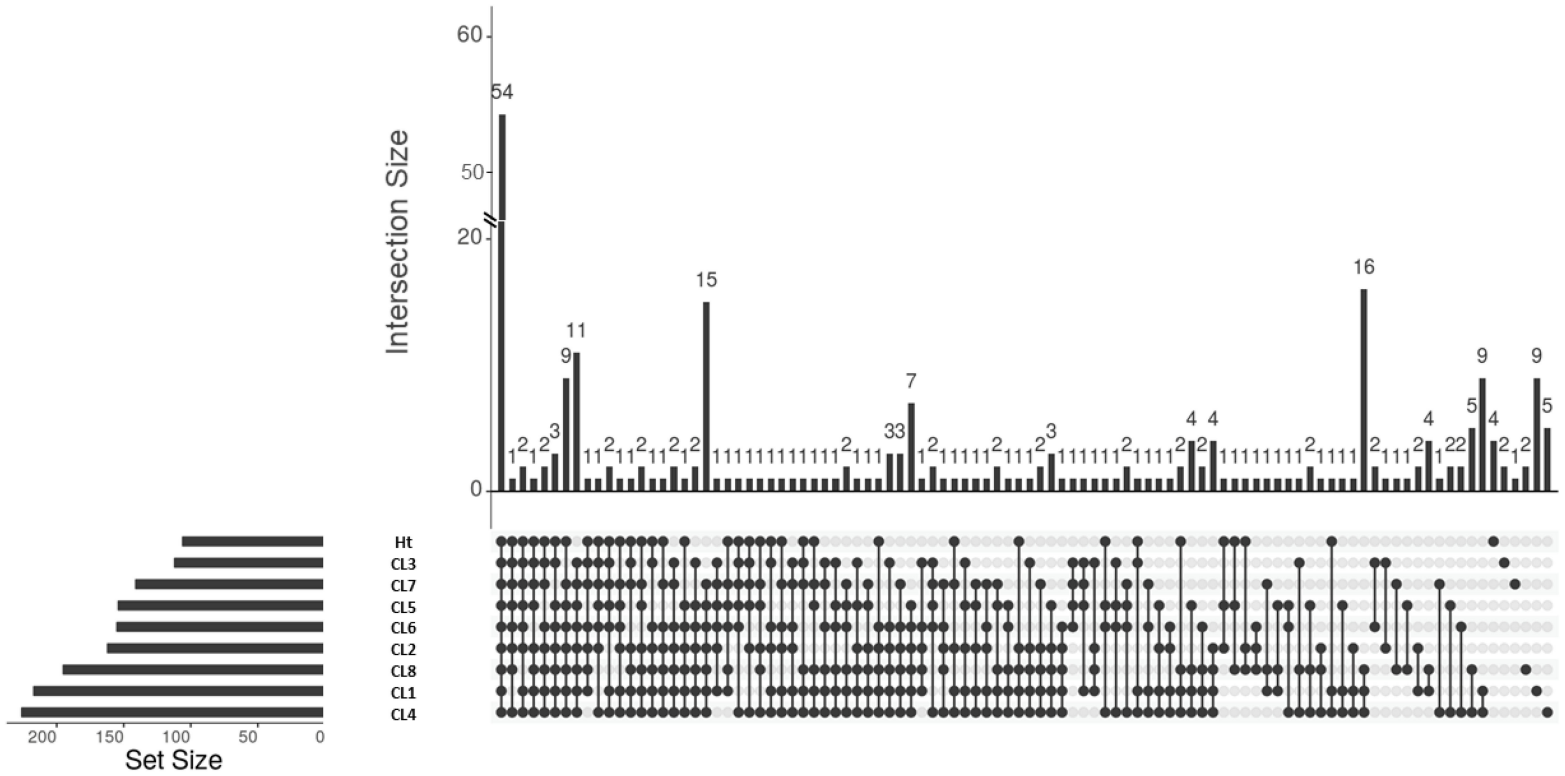
UpSet plot representing the shared and unique proteins present in *L. mexicana* clones and Ht. The legend is found below the plot, where each dot represents the presence of the proteins in that sample and the line representing that these proteins are shared between all samples with a dot. Samples are ordered from least unique proteins to most.

A total of 253 proteins were identified across all 8 *L. mexicana* clonal EVs and the Ht EVs. These proteins were analyzed using Scaffold 4 (**Supplementary Table 1**). Unique and shared proteins between the 8 *L. mexicana* clones and Ht *L. mexicana* was visualized using an UpSet plot (**Figure 3**). Surprisingly few proteins were identified in EVs collected from the Ht *L. mexicana* culture, having the lowest number of individual proteins identified, followed by CL3 and 7. Ht *L. mexicana* and CL3 also had the most proteins uniquely absent, at 11 and 9 respectively, as well as sharing 15 proteins absent from all other groups (**Table 2**). This was unexpected considering that the Ht *L. mexicana* was the original progenitor strain, and thus we expected its EVs to contain most if not all proteins found in other clonal EVs. Surprisingly, despite CL7 having the next fewest individual proteins identified, there were only 3 proteins, long-chain-fatty-acid-CoA ligase, surface protein amastin, and amastin-like protein, uniquely absent from CL7 compared to all other groups, showing that there was strong overlap between CL7 and other clones altogether. CL8 EVs also uniquely lacked a cytochrome C protein, which is an important cell respiratory component.

**Table 2:** Proteins categorized based on being present in only a single *L. mexicana* clone (Top) or missing from only a single *L. mexicana* clone (Bottom).

Unique to	Protein name	Accession number	Molecular weight	Function
**CL1**	Protein disulfide-isomerase	E9AUD1_LEIMU	52 kDa	Catalyses protein folding
	Putative carboxypeptidase	E9B493_LEIMU	57 kDa	Metallocarboxypeptidase
	Fructose-bisphosphate aldolase	E9ASP6_LEIMU	41 kDa	Glycolysis
	Putative ATP-dependent Clp protease subunit, heat shock protein 100 (HSP100)	E9ALU6_LEIMU	97 kDa	ATP binding
	Putative short chain dehydrogenase	E9B601_LEIMU	25 kDa	Oxidoreductase
	Putative lipophosphoglycan biosynthetic protein	E9AM02_LEIMU	87 kDa	Chaperone
**CL3**	S-methyl-5’-thioadenosine phosphorylase	E9AKI9_LEIMU	33 kDa	L-methionine biosynthesis salvage pathway
**CL4**	Conserved TPR domain protein	E9B146_LEIMU	45 kDa	HSP-binding
	Kinesin-like protein	E9AR52_LEIMU	105 kDa	ATP-dependent microtubule motor
**CL8**	Putative asparagine synthetase a (Putative aspartate-ammonia ligase)	E9AY61_LEIMU	40 kDa	Asparagine biosynthesis
	Putative serine/threonine-protein kinase	E9AVE6_LEIMU	90 kDa	Protein kinase
**Ht**	Amastin-like surface protein-like protein	E9B0L6_LEIMU	25 kDa	Integral membrane protein
	Putative zinc transporter	E9B012_LEIMU	47 kDa	Transmembrane zinc transporter
	GTP-binding nuclear protein	E9AXM1_LEIMU	24 kDa	Protein transport
	Transmembrane 9 superfamily member	E9ALR2_LEIMU	77 kDa	Signal transmembrane helix

Missing from	Protein name	Accession number	Molecular Weight	Function
**CL1**	Putative 3’-nucleotidase/nuclease	E9ANW7_LEIMU	41 kDa	Endonuclease
**CL3**	Putative myosin heavy chain	E9B3G5_LEIMU	119 kDa	Actin binding motor
	Putative ecotin	E9AQ49_LEIMU	41 kDa	Serine-type endopeptidase inhibitor
	Putative casein kinase	E9B5Y1_LEIMU	40 kDa	Serine/threonine kinase
	Putative calpain-like cysteine peptidase	E9AK26_LEIMU	89 kDa	Calcium-dependent cysteine-type endopeptidase
	Proteasome regulatory ATPase subunit	E9ATM0_LEIMU	46 kDa	Proteasome-activating
	Glyceraldehyde-3-phosphate dehydrogenase	E9B170_LEIMU	39 kDa	Glycolysis
	Putative proteophosphoglycan ppg3 (Fragment)	E9B5T5_LEIMU	120 kDa	Extracellular matrix
	GDP-mannose pyrophosphorylase	Q9BLW4_LEIME	41 kDa	Mannose-1-phosphate guanylyltransferase
	Putative small GTP-binding protein Rab1	E9AYX8_LEIMU	22 kDa	GTPase
**CL5**	Clathrin heavy chain	E9AST4_LEIMU	191 kDa	Intracellular/vesicle-mediated transport
	Putative small GTP-binding protein Rab11	E9ANA1_LEIMU	23 kDa	GTP binding
**CL6**	Putative c2 domain protein	E9B1N1_LEIMU	30 kDa	Calcium/lipid-binding domain
**CL7**	Putative long-chain-fatty-acid-CoA ligase	E9AJD9_LEIMU	78 kDa	Long-chain fatty acid-CoA ligase
	Amastin-like protein	E9B4S0_LEIMU	21 kDa	Integral membrane protein
	Putative surface protein amastin	E9B0L4_LEIMU	21 kDa	Integral membrane protein
**CL8**	Putative cytochrome c	E9AQU2_LEIMU	12 kDa	Electron transport
**Ht**	Pyruvate kinase	E9B5P1_LEIMU	54 kDa	Glycolysis
	2,3-bisphosphoglycerate-independent phosphoglycerate mutase	E9AUA1_LEIMU	61 kDa	Glycolysis
	Heat shock protein 70-related protein	E9AYA3_LEIMU	71 kDa	ATP binding
	Putative ABC transporter	E9AYZ8_LEIMU	203 kDa	ATPase-coupled transmembrane transporter
	Putative seryl-tRNA synthetase	E9ANF6_LEIMU	53 kDa	Seryl-tRNA aminoacylation
	Trypanothione reductase	E9AKE1_LEIMU	53 kDa	Glutathione reductase equivalent
	Serine/threonine-protein phosphatase	E9AKB4_LEIMU	72 kDa	Serine/threonine phosphatase
	Putative adenosine kinase	E9B0L7_LEIMU	37 kDa	AMP biosynthesis salvage pathway
	Kinesin-like protein	E9ARX7_LEIMU	94 kDa	Microtubule-based movement
	Proteasome subunit alpha type	E9AVH9_LEIMU	27 kDa	Ubiquitin-dependent hydrolase
	Putative Qc-SNARE protein	E9AM81_LEIMU	24 kDa	Vesicle-mediated transport

### EV protein abundance varies between different *L. mexicana* clones

**Figure 4:**
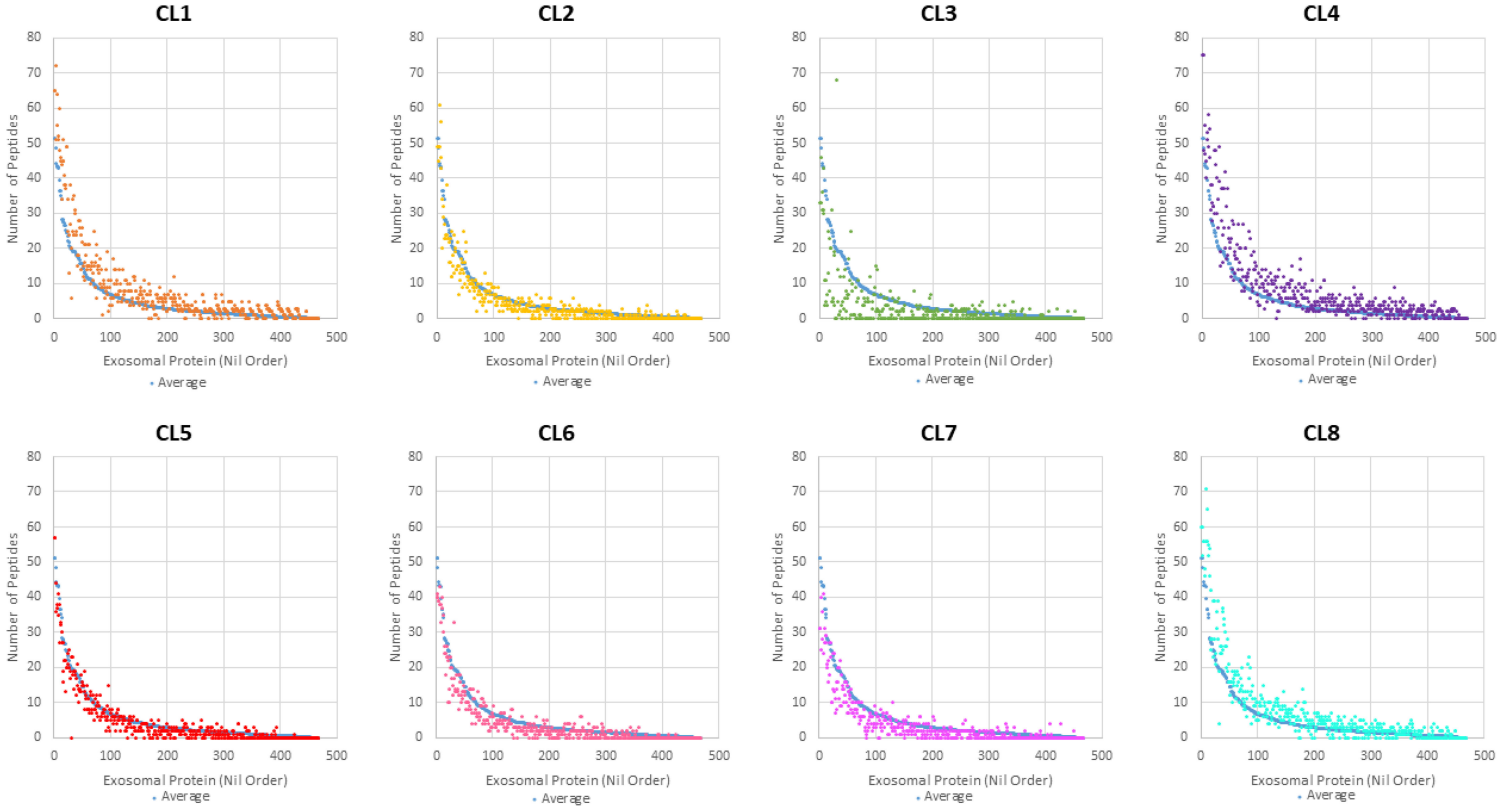
Overall protein abundance of each *L. mexicana* clone was plotted and compared, with the blue dots representing the baseline. Each dot represents an individual protein and the spectrum count associated with that protein.

**Figure 5:**
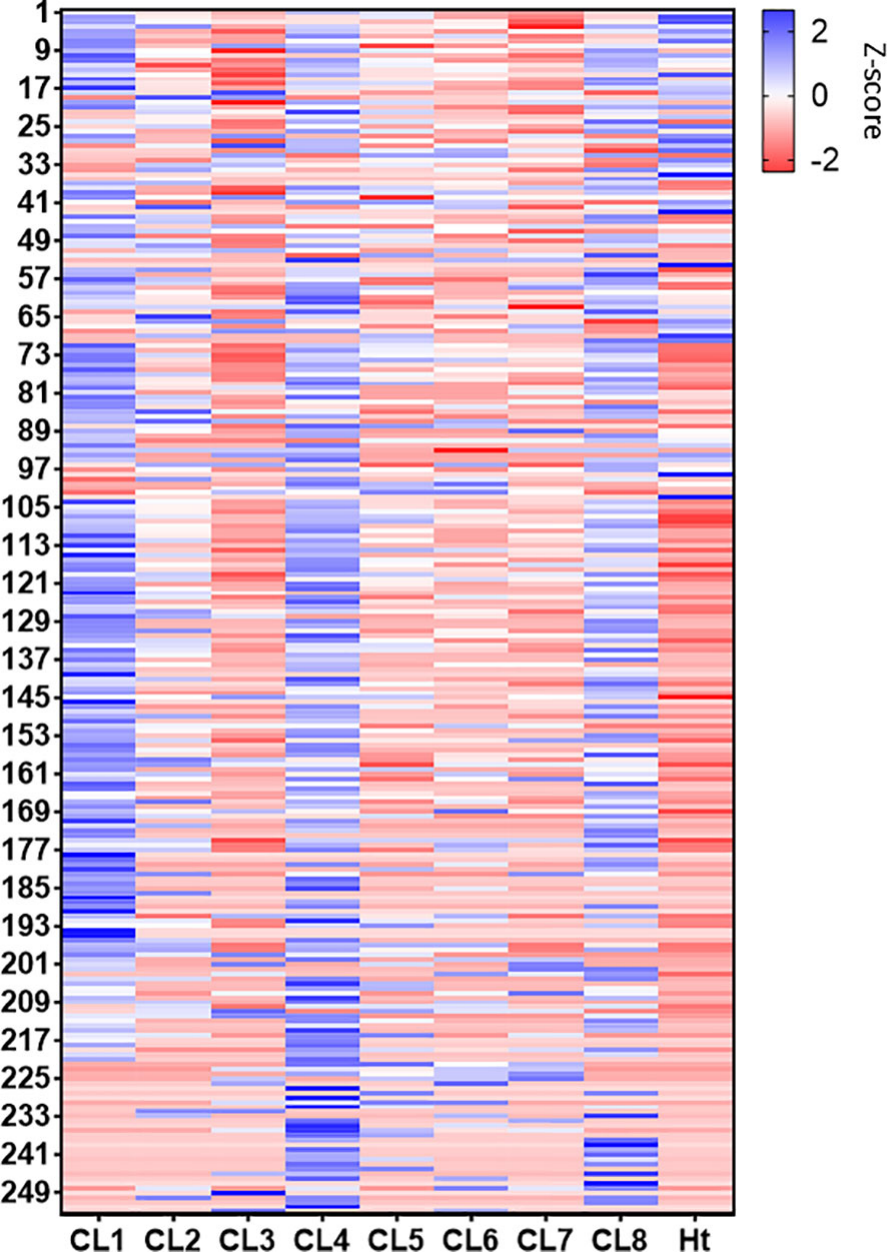
Heatmap generated representing all proteins identified (253) in the *L. mexicana* clones and Ht using Z-score. For protein identities, see **Supplementary Materials** (**Supplementary Table 1**).

**Figure 6:**
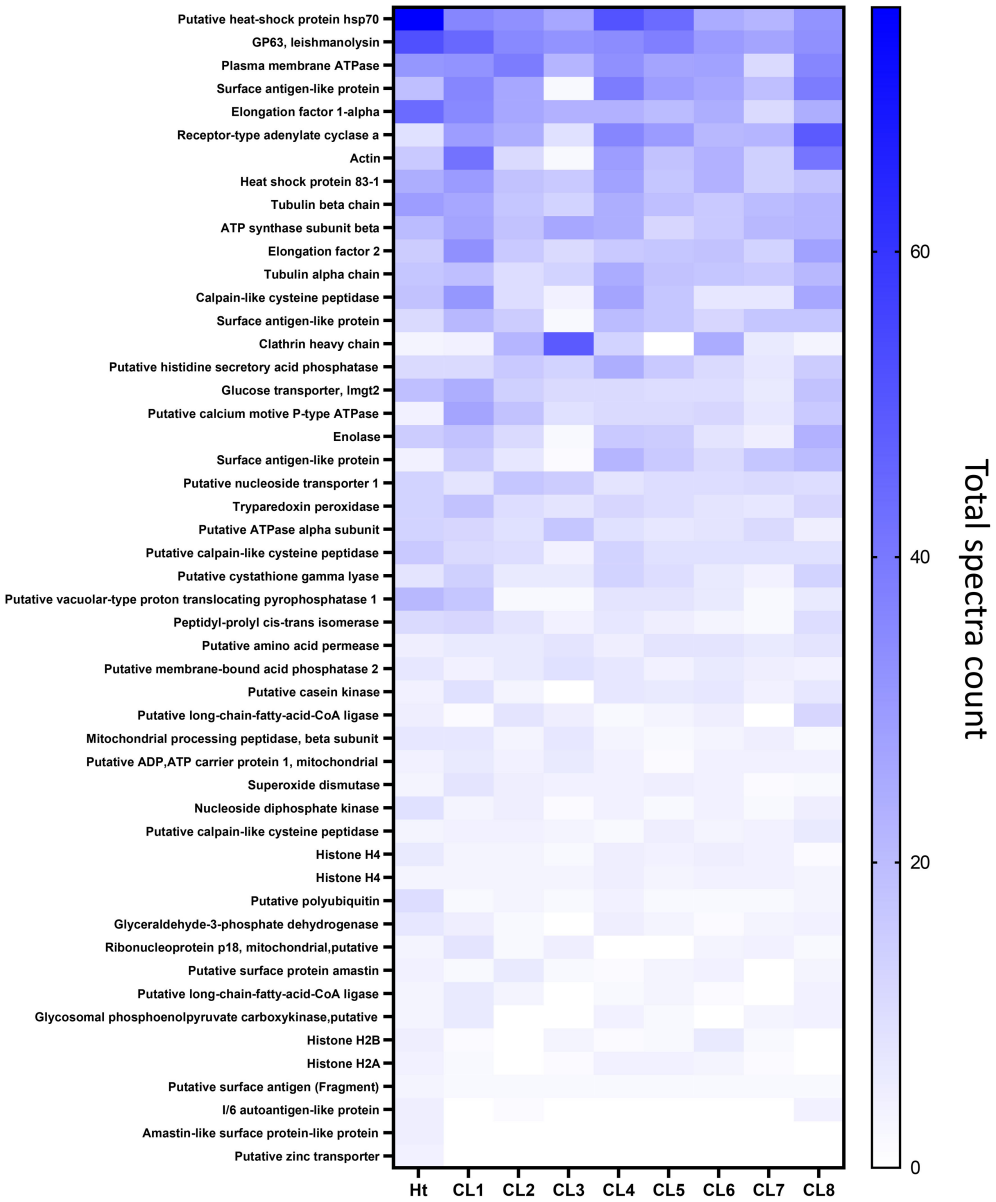
Heatmap generated representing the top 50 most abundant proteins identified in the *L. mexicana* clones and Ht using total spectrum count and ordered by Ht.

Overall protein abundance was measured in total spectra score using scaffold and compared between the 8 clones (**Figure 4**). From this comparison, it is evident that CL1, 4, and 8 EVs generally contain more proteins compared to other clones, with the blue line representing average total spectrum count of all clones. This higher protein abundance is especially clear when representing this data using a Z-score heatmap with all 253 identified proteins (**Figure 5**). To better display typically high abundance proteins found in *Leishmania*, a heatmap was also generated with the top 50 most abundant proteins across all clonal and Ht EVs, with this limited scope also allowing visualization of total spectra rather than Z-score (**Figure 6**). CL7 EVs contained the lowest amount of GP63, HSP70, HSP83-1, plasma membrane ATPase, EF1a, and EF2 (**Figure 6**; **Supplementary Table 1**). CL3 EVs had lower overall protein abundance but a significantly higher quantity of clathrin heavy chain. CL1 EVs contained the highest amount of actin, GP63, EF1a, and EF2 amongst the clones. Although Ht EVs contained fewer unique proteins, overall protein abundance was higher than EVs from other *L. mexicana* clones, having the highest amount of HSP70, GP63, EF1a, and tubulin beta. CL8 EVs contained the highest amount of receptor-type adenylate cyclase A. The combined low abundance and low number of proteins identified in CL3 EVs suggested that there was material loss during the processing or analysis of this sample, resulting in its exclusion from further analysis. However, CL3 EVs contained by far the most clathrin heavy chain despite their low overall protein abundance.

### Overall protein function does not vary between clone EVs

**Figure 7:**
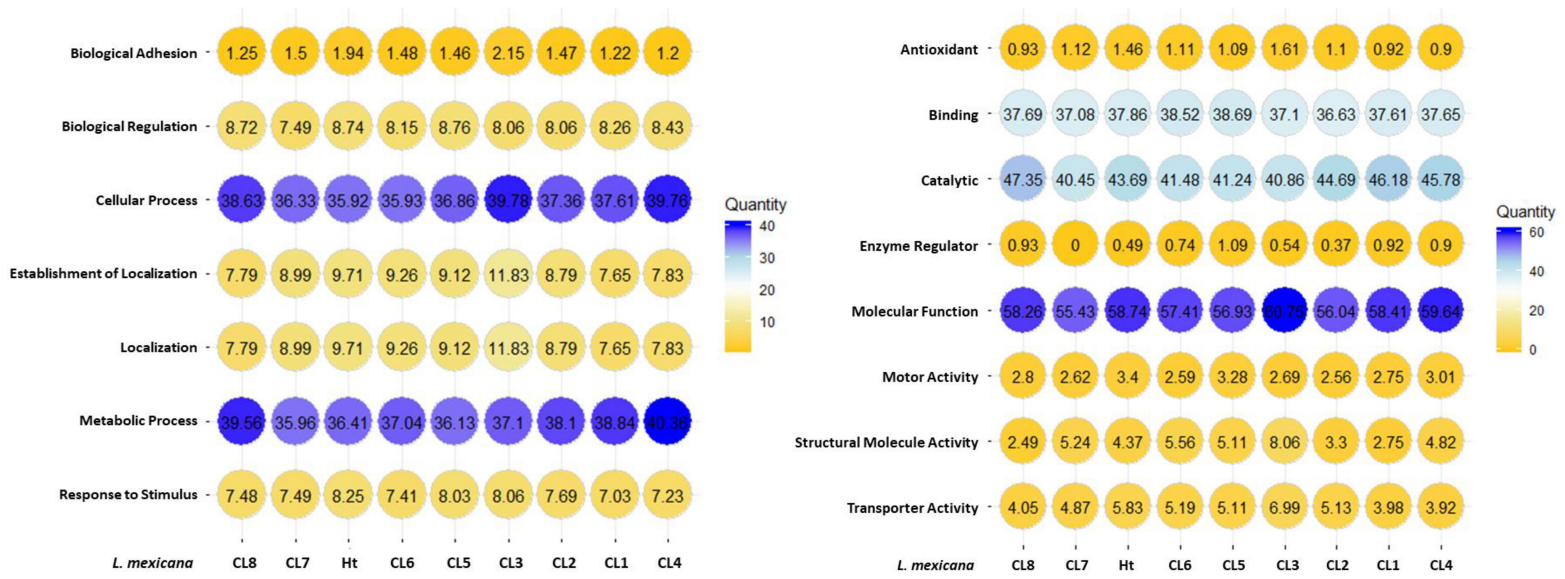
Gene ontology performed for searching for terms related to biological processes and molecular functions. Number present in circles represents the percentage of proteins of that specific category (Eg. Biological adhesion) compared to all proteins identified in the sample. The color legend indicates the spectrum count.

**Figure 8:**
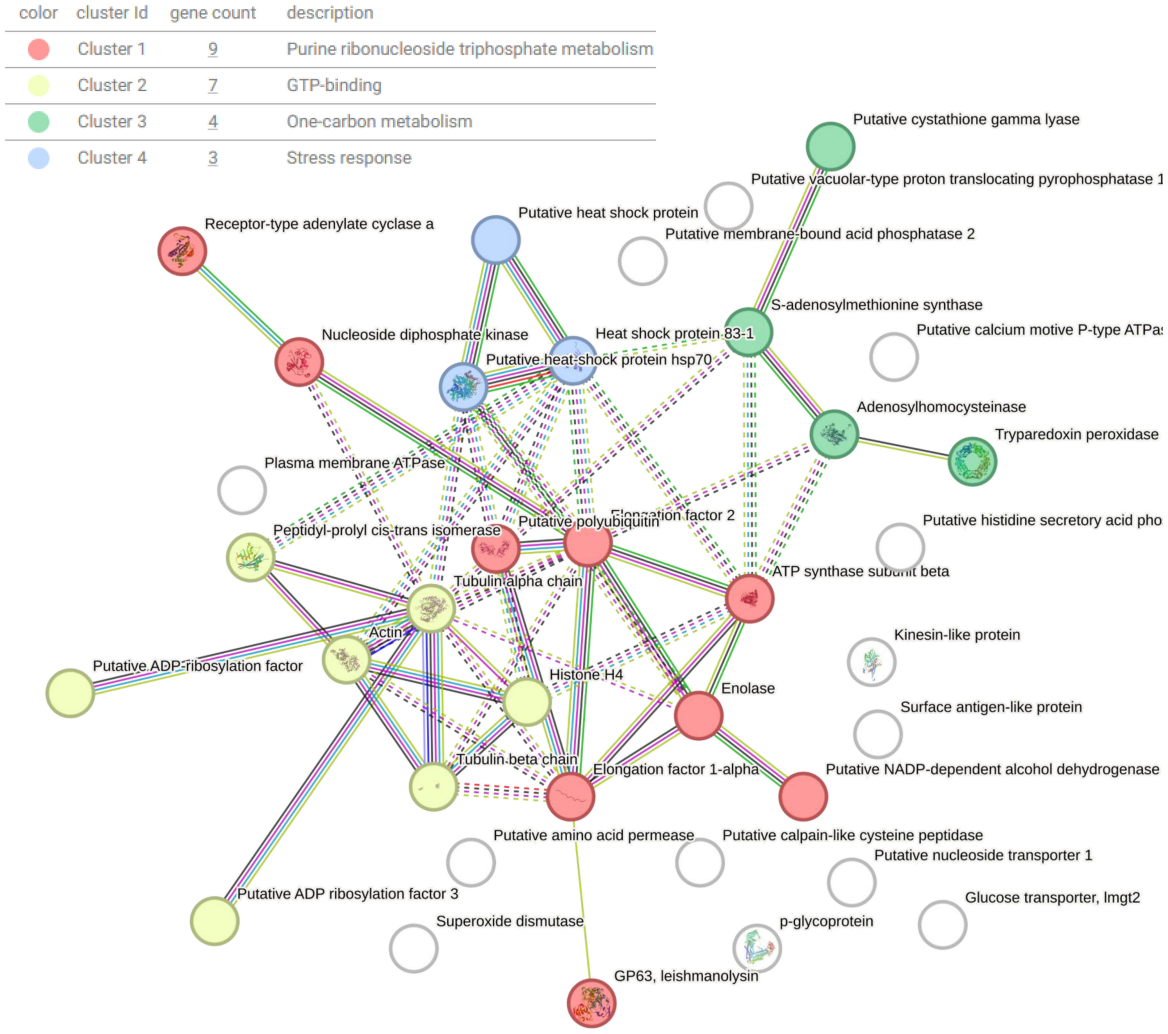
STRING diagram representing interactions between shared proteins present in all EVs isolated from *L. mexicana* clones and Ht. Proteins are clustered based on function using k-means clustering. Lines represent a direct relationship between proteins, with dotted lines representing the edge of a cluster.

Gene ontology (GO) annotations were attributed using Panther and represented using percentages for each clone and Ht *L. mexicana* EVs (**Figure 7**). Annotations for biological processes are visualized on the left and molecular function on the right. Cellular and metabolic processes were the most common biological processes annotated to *L. mexicana* EVs. Although localization and establishment of localization appeared as distinct categories during the GO search, these categories overlapped entirely. When searching by molecular function, there was strong overlap with the very broad molecular function category which encompassed almost all proteins with a hypothesized mechanism of action. There was no significant variation in EV protein content when looking at these broader GO terms between the different clones compared. However, proteins related to enzyme regulation were entirely absent in CL7 EVs. The 51 shared proteins between all groups are visualized in a STRING diagram to show hypothesized protein interactions (**Figure 8**; **Table 3**). We found 4 functional protein clusters shared between all *L. mexicana* clones and Ht *L. mexicana*: purine ribonucleoside triphosphate metabolism (Atayde et al., 2015), GTP-binding (Silverman et al., 2010a), one-carbon metabolism (Mann et al., 2021), and stress response (Dong et al., 2019). Several *Leishmania* virulence factors, like GP63, EF1α, and enolase, fall under the purine ribonucleoside triphosphate metabolism cluster, while important cytoskeletal proteins, like actin and tubulin, fall under the GTP-binding cluster.

**Table 3:** All proteins shared between all *L. mexicana* clones and Ht. Proteins were present with at least 2 peptides and 95% confidence, as determined by Scaffold4.

Protein name	Accession number	Function
Putative heat-shock protein hsp70	E9B099_LEIMU	Heat/stress response
Elongation factor 1-alpha	E9ARD0_LEIMU	Translation elongation factor GTPase
Plasma membrane ATPase	E9ART6_LEIMU	Plasma membrane proton transport
GP63, leishmanolysin	E9AN57_LEIMU	Virulence and cell survival
Receptor-type adenylate cyclase a	E9ARD7_LEIMU	Cell signalling and survival
Elongation factor 2	E9ASD6_LEIMU	Translation elongation factor GTPase
Surface antigen-like protein	E9AK01_LEIMU	EGF-like domain
Actin	C6KJD5_LEIME	Cytoskeletal structure
Heat shock protein 83-1	E9B3L2_LEIMU	Heat/stress response
Calpain-like cysteine peptidase	E9AUQ7_LEIMU	Calcium-dependent cysteine-type endopeptidase
Tubulin beta chain	E9AMJ9_LEIMU	Cytoskeletal structure
p-glycoprotein	E9B4S1_LEIMU	Drug resistance
ATP synthase subunit beta	E9AXJ6_LEIMU	Mitochondrial ATP synthesis
Enolase	E9APW3_LEIMU	Glycolysis
Tubulin alpha chain	E9AP62_LEIMU	Cytoskeletal structure
Surface antigen-like protein	E9AKJ6_LEIMU	EGF-like domain
Putative calcium motive P-type ATPase	E9B686_LEIMU	Cellular ion homeostasis
Putative histidine secretory acid phosphatase	E9AU82_LEIMU	Acid phosphatase
Kinesin-like protein	E9AKI2_LEIMU	Microtubule motor activity
Putative heat shock protein	E9ARS1_LEIMU	Heat/stress response
Glucose transporter, lmgt2	E9AU63_LEIMU	Cellular glucose homeostasis
Surface antigen-like protein	E9AKM8_LEIMU	EGF-like domain
Tryparedoxin peroxidase	E9AQA6_LEIMU	Hydroperoxide resistance
Putative nucleoside transporter 1	E9ASW8_LEIMU	Transmembrane nucleoside transport
Putative calpain-like cysteine peptidase	E9AUS1_LEIMU	Calcium-dependent cysteine-type endopeptidase
Putative cystathione gamma lyase	E9B6K2_LEIMU	Cysteine biosynthesis
Peptidyl-prolyl cis-trans isomerase	E9AXG9_LEIMU	Protein folding
Putative ATPase alpha subunit	E8NHQ7_LEIMU	Mitochondrial ATP synthesis
Putative vacuolar-type proton translocating pyrophosphatase 1	E9B1S0_LEIMU	Plasma membrane proton transport
Putative amino acid permease	E9AYW8_LEIMU	Plasma membrane amino acid transport
Putative ADP ribosylation factor 3	E9ALY6_LEIMU	Initiates microtubule polymerization
Nucleoside diphosphate kinase	E9B376_LEIMU	Triphosphate biosynthesis
Putative NADP-dependent alcohol dehydrogenase	E9AW40_LEIMU	Alcohol dehydrogenase
Putative membrane-bound acid phosphatase 2	E9AT36_LEIMU	Acid phosphatase
Putative calpain-like cysteine peptidase	E9AUR8_LEIMU	Calcium-dependent cysteine-type endopeptidase
Putative serine/threonine-protein kinase (Putative protein kinase)	E9AXW8_LEIMU	Protein kinase
Surface antigen-like protein	E9AK03_LEIMU	EGF-like domain
Putative ADP,ATP carrier protein 1, mitochondrial	E9ARX1_LEIMU	Mitochondrial ATP/ADP transport
Putative tyrosine aminotransferase	E9AT13_LEIMU	Amino acid metabolism
Mitochondrial processing peptidase, beta subunit,putative,metallo-peptidase, Clan ME, Family M16	E9B617_LEIMU	Mitochondrial matrix
Adenosylhomocysteinase	E9ATH3_LEIMU	Homocysteine biosynthesis
Putative ADP-ribosylation factor	E9B278_LEIMU	GTPase
Putative polyubiquitin	E9AMU1_LEIMU	Proteosome recruitment
Superoxide dismutase	E9B2V9_LEIMU	Free radical resistance
S-adenosylmethionine synthase	E9B1C6_LEIMU	Adenosylmethionine biosynthesis
Putative calpain-like cysteine peptidase (Putative cysteine peptidase, clan ca, family c2)	E9APT0_LEIMU	Calcium-dependent cysteine-type endopeptidase
Putative surface antigen (Fragment)	E9ANZ9_LEIMU	EGF-like domain
Histone H4	E9ASB9_LEIMU	Nucleosome core component
Histone H4	E9AKM9_LEIMU	Nucleosome core component

### Vesicles isolated from selected clones exhibit varied infectious capability compared to parasite virulence alone

**Figure 9:**
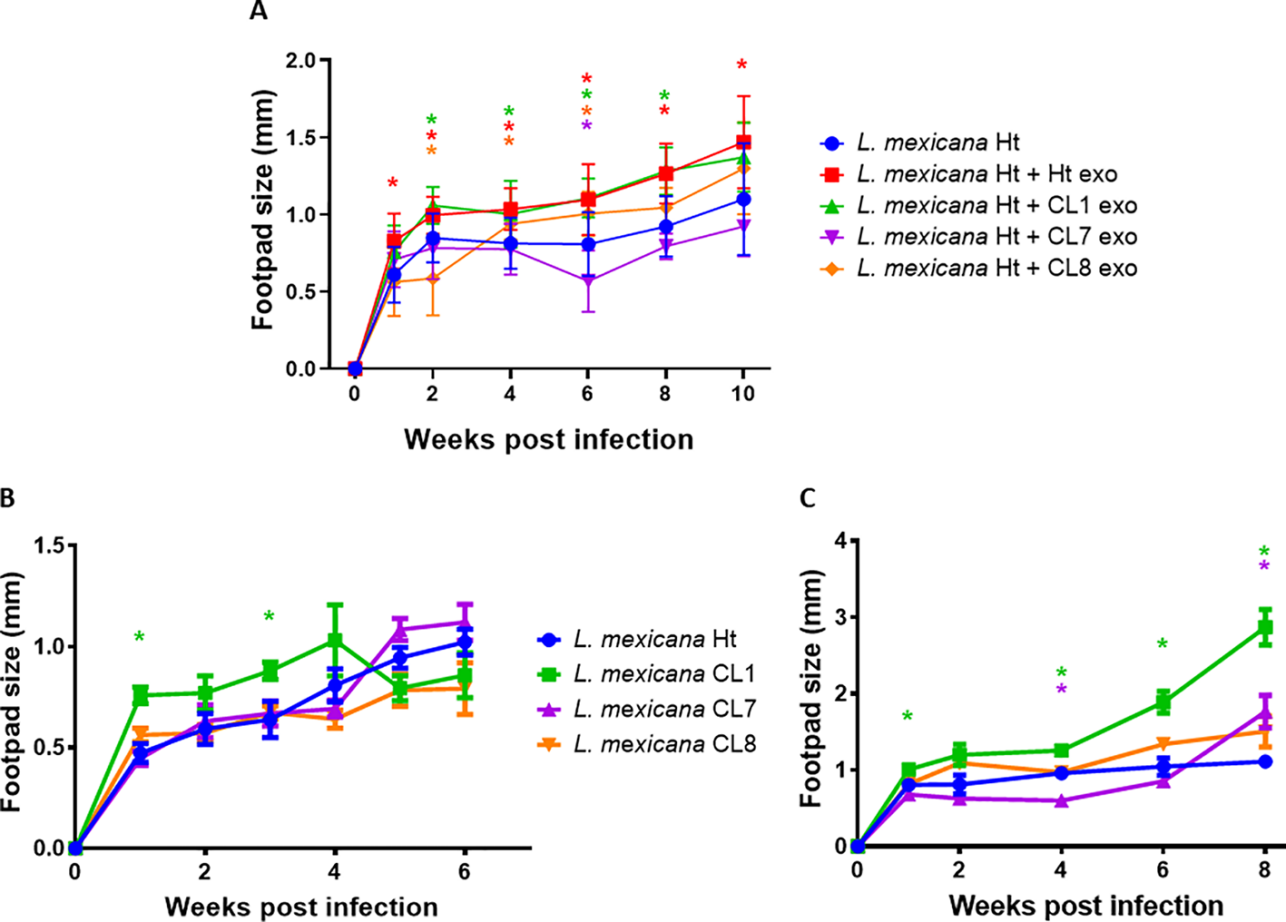
Graphs depicting C57BL6 **(A)** and BALB/C **(C)** mice footpad infections performed with Ht *L. mexicana* supplemented with CL1, 7 and 8 EVs infecting C57BL6 mice **(A)** or Ht, CL1, 7, and 8 *L. mexicana* parasite alone infecting C57BL6 mice **(B)** or Balb/C mice **(C)**. Significance (P<0.05) is denoted by "*", comparing to the Ht *L. mexicana* infection alone, with color indicating the significant infection corresponding to the legend.

The infectious capability of *L. mexicana* clone EVs was tested using a C57BL6 murine footpad infection model, measuring the subsequent footpad swelling to determine infection severity (**Figure 9A**). The co-inoculation of Ht *L. mexicana* along with Ht and CL1 EVs significantly worsened the footpad infection compared to Ht *L. mexicana* alone at all time points measured, consistently causing a 0.2mm increase in footpad swelling at the start of infection and increasing to a 0.3mm difference by week 6 of the infection. CL8 EVs co-inoculation actually reduced footpad swelling at the start of infection compared to Ht and CL1 EVs, even showing an inhibitory effect at week 2. However, the infection proceeded as expected from week 3 onwards, causing around a 0.1mm increase in swelling compared to Ht alone. Strikingly, CL7 EVs did not cause significantly increased footpad swelling compared to Ht alone. The infectious capability of the *L, mexicana* clone parasites was tested as well, omitting EV co-inoculation (**Figure 9B**). Surprisingly, the infectious capability of the parasites themselves differed from what was seen with the EVs. Although CL1 EVs did not augment infection more than Ht EVs, CL1 parasites alone caused more severe infection during the first 4 weeks compared to Ht parasites, although footpad swelling was only significantly greater at weeks 1 and 3. The other selected clones did not cause significantly altered infection overall compared to Ht. We also assessed parasite infectious capability using Balb/c mice (**Figure 9C**). The resulting footpad swelling was significantly greater compared to C57BL6 infection. With the Balb/c model, we found that all *L. mexicana* clones caused similar infection to Ht *L. mexicana* with the exception of CL1, which caused significantly more footpad swelling at almost all timepoints measured (weeks 1, 4, 6, and 8 post-infection). CL7 only caused reduced footpad swelling at week 4.

## Discussion

The heterogeneity of EVs is a source of great discussion within the field of EVs, leading to great interest in isolating and comparing subpopulations of EVs secreted by a single population of cells or organisms. By isolating clonal colonies of *L. mexicana*, a highly heterogenous species of *Leishmania*, we are attempting to similarly isolate different populations of EVs from a single progenitor strain of *L. mexicana.* When comparing clonal *L. mexicana* EVs based on their physical properties, they are indistinguishable from one another. There are slight variations in mean diameter and size distribution, but such variations are common between different preparations of EVs rather than being indicative of an overall trend. The parasites themselves are also indistinguishable in terms of morphology and growth rate.

However, proteomic analysis reveals that there is significant variation in the EV protein content of each *L. mexicana* clone. With over 200 individual proteins identified across all groups, only 51 were shared between all groups. As evident from the STRING diagram, these are all important *Leishmania* proteins that are typically found in *Leishmania* EVs, relating to stress response, virulence, cytoskeleton, and metabolism (Nugent et al., 2004; Besteiro et al., 2007; da Silva Lira Filho et al., 2024). Surprisingly, Ht EVs had the fewest individual proteins identified, despite having comparable protein abundance to other groups. This may be due to the inherent heterogeneity of the Ht EVs, with the large amount of unique low-abundance proteins causing the equivalent of noise and preventing their detection by mass spectrometry, especially when compounded with the relatively low starting protein quantity. Such difficulties are commonly reported when attempting proteomic analysis on samples with only a few highly abundant proteins (Echan et al., 2005; Bellei et al., 2011).

On the other hand, CL3 EVs had fewer identified proteins as well as low protein abundance, which could suggest material loss during sample preparation and processing for mass spectrometry. Interestingly, despite the low overall protein abundance, CL3 EVs had the greatest abundance of clathrin heavy chain, which has been implicated in EV uptake by cells (Tian et al., 2014). However, many other such uptake mechanisms have been reported that are clathrin-independent (Svensson et al., 2013; Costa Verdera et al., 2017), and the high abundance of clathrin may even suggest CL3 EVs originate from a unique pathway compared to EVs from the other clones which contain much less clathrin, explaining the lower quantity of collected material. Regardless, due to the low amount of material, CL3 EVs were not used in further experiments.

CL1, 4, and 8 EVs had higher overall protein abundance compared to other clones, suggesting potential enrichment of protein cargo in the EVs of these clones. Of particular interest were CL1 EVs, which contained the highest amount of GP63, EF1α, and EF2, all important *Leishmania* virulence factors (Nandan et al., 2002; Kushawaha et al., 2011; Isnard et al., 2012). These differences are important due to the limited size of EVs, which means cargo sorted into the EVs must be selected by some mechanism and such minute differences can heavily impact EV physiological function (Kalluri and LeBleu, 2020), specifically their ability to enhance infection in the case of these *Leishmania* EVs. However, these differences are relatively small when considering the variance that is inherent to mass spectrometry-based proteomic analysis. Indeed, analysis using gene ontology did not reveal any significant broad functional differences between *L. mexicana* clonal EVs.

The most striking differences between the *L. mexicana* clones were apparent when testing the infectious capabilities of the EVs. Ht *L. mexicana* EVs were able to augment infection as expected, *L. mexicana* CL7 EVs completely lost their infection-enhancing properties and failed to significantly augment infection at any time point. This reduced virulence was not observed when comparing the infectious capabilities of the clonal *L. mexicana* alone without EV co-inoculation in both C57BL6 and Balb/c mice, with Balb/C infection resulting in greater footpad swelling as expected (Sacks and Noben-Trauth, 2002). This is consistent with what was observed in *L. amazonensis*, where EVs containing high amounts of GP63 did not significantly enhance infection compared to WT *L. amazonensis* EVs, but GP63low EVs did not significantly augment infection compared to *L. amazonensis* alone (da Silva Lira Filho et al., 2021). Additionally, it has been reported that exosomal GP63 modulates hepatic lipid metabolism and lowers serum cholesterol through the cleavage of DICER1, which leads to the inhibition of miR-122 (Ghosh et al., 2013). This further demonstrates that GP63-loaded EVs are specifically capable of modulating host cells. However, the difference in GP63 quantity observed in this study is much lower comparatively, so this explanation alone is not sufficient in explaining the loss of infectious capability of CL1 EVs. Of particular interest, CL7 EVs were also deficient in surface protein amastin and CL8 EVs were deficient in cytochrome C. Cytochrome C is an important oxidase that allows *Leishmania* to adapt to human macrophage environment and amastin is a classical *Leishmania* surface virulence factor, important for parasite survival in the sandfly vector as well as infection (Cardenas et al., 2015; de Paiva et al., 2015). However, as we observed, the lack of cytochrome C did not result in any reduced infectivity in either CL8 parasites or its EVs.

In conclusion, although the EVs secreted by different *L. mexicana* clones are physically indistinguishable from each other, proteomic analysis reveals that they each have unique protein content when considering the overall presence/absence of individual proteins as well as protein abundance. These differences may lead to varying infectious capabilities of the clonal *L. mexicana* EVs, as we observed when infecting C57BL6 footpads with CL1, 7, and 8 EVs, comparing to Ht *L. mexicana*, with CL7 EVs losing their infection-augmenting properties. Comparatively, the clonal parasites alone caused mostly consistent levels of footpad swelling. This shows that EVs have unique properties compared to the parasite that secreted them. Despite each clonal *L. mexicana* being derived from the same *L. mexicana* Ht culture, their EVs have notable differences in protein content and infectious capability. To validate this work, we would like to sequence the *L. mexicana* clones used in this study, especially CL1, CL7, and CL8, in order to verify the genetic homogeneity of each clone as well as identify the genetic variations that may lead to the observed differences in infectious capability. This would open the up further gene manipulation studies. It will also be prudent to perform further immunoblotting to validate the proteomic data.

## Data Availability

Original proteomic datasets are available in the publicly accessible PRIDE database. This data can be found at DOI 10.6019/PXD058429 under accession number PXD058429.
