# Supplementary material for: Unique Leishmania mexicana clones secrete populations of extracellular vesicles with unique protein profile and variable infectious capability
Source: Front Cell Infect Microbiol. 2024 Dec 5;14:1443262. doi: 10.3389/fcimb.2024.1443262 (PMC11655471; doi:10.3389/fcimb.2024.1443262)
Supplement: Supplementary file 1 [file DataSheet1.docx]

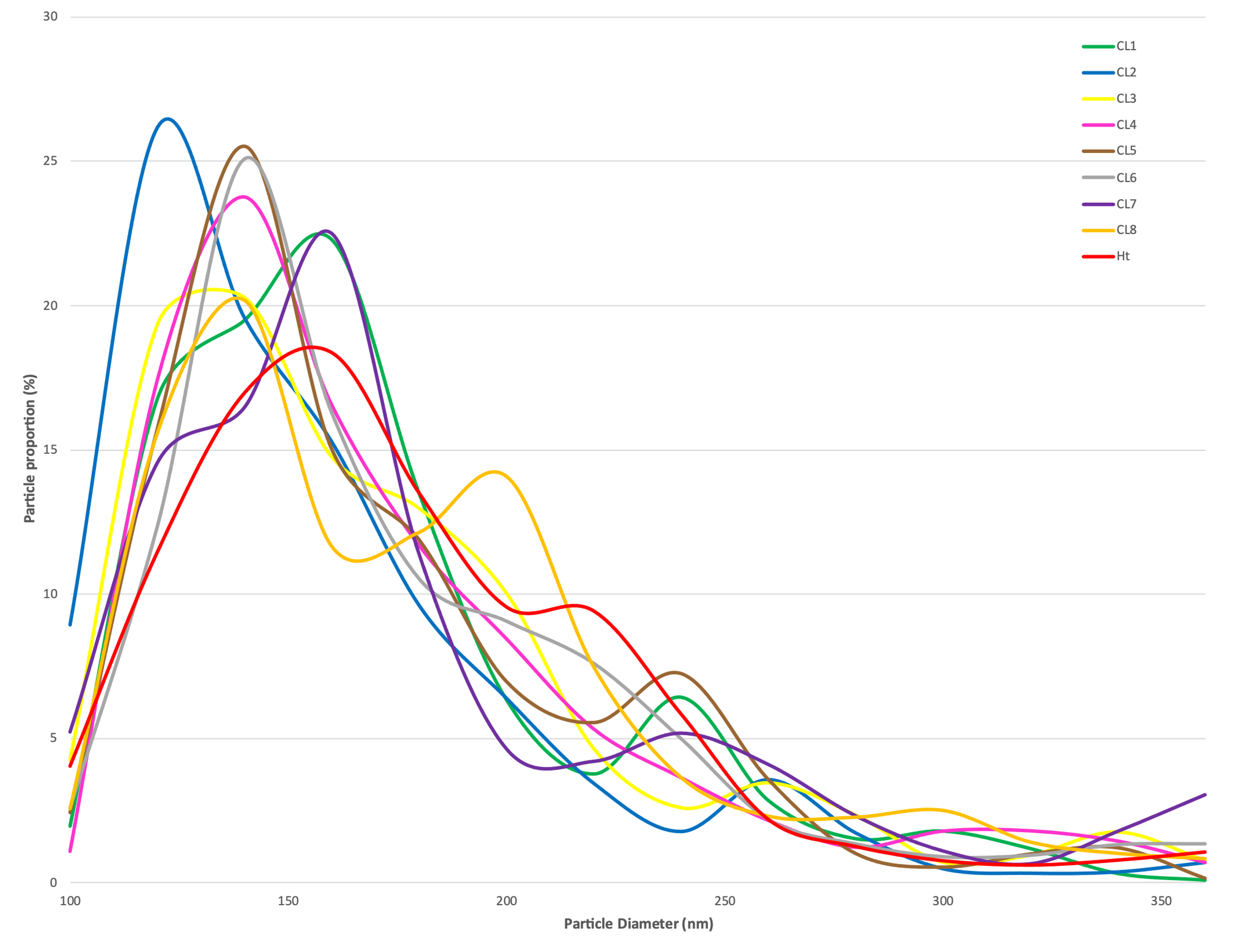


Supplemental Figure 1. Proportional size distribution profile of all EVs isolated from *L. mexicana* clones and Ht *L. mexicana* superimposed to directly compare their size distribution. Proportion was calculated based on the total particle concentration of each separate clone/Ht.
