# Supplementary material for: Unique Leishmania mexicana clones secrete populations of extracellular vesicles with unique protein profile and variable infectious capability
Source: Front Cell Infect Microbiol. 2024 Dec 5;14:1443262. doi: 10.3389/fcimb.2024.1443262 (PMC11655471; doi:10.3389/fcimb.2024.1443262)
Supplement: Supplementary file 3 [file Table2.docx]

|  | **Identified Protein** | **Accession Number** | **Molecular Weight (kDa)** | **CL1** | **CL2** | **CL3** | **CL4** | **CL5** | **CL6** | **CL7** | **CL8** | **Ht** |
| --- | --- | --- | --- | --- | --- | --- | --- | --- | --- | --- | --- | --- |
| **1** | Putative heat-shock protein hsp70 | E9B099_LEIMU [2] | 71 | 36 | 33 | 26 | 51 | 44 | 25 | 22 | 32 | 76 |
| **2** | GP63, leishmanolysin | E9AN57_LEIMU [5] | 64 | 45 | 35 | 32 | 34 | 38 | 30 | 27 | 33 | 52 |
| **3** | Elongation factor 1-alpha | E9ARD0_LEIMU | 49 | 35 | 26 | 23 | 23 | 20 | 24 | 11 | 24 | 44 |
| **4** | Plasma membrane ATPase | E9ART6_LEIMU [2] | 108 | 32 | 39 | 22 | 33 | 27 | 28 | 11 | 36 | 31 |
| **5** | Tubulin beta chain | E9AMJ9_LEIMU [2] | 50 | 26 | 17 | 13 | 24 | 19 | 16 | 20 | 22 | 29 |
| **6** | Heat shock protein 83-1 | E9B3L2_LEIMU [2] | 81 | 30 | 18 | 16 | 28 | 17 | 23 | 14 | 18 | 24 |
| **7** | Putative vacuolar-type proton translocating pyrophosphatase 1 | E9B1S0_LEIMU | 84 | 17 | 2 | 2 | 8 | 8 | 6 | 2 | 6 | 21 |
| **8** | ATP synthase subunit beta | E9AXJ6_LEIMU | 56 | 27 | 18 | 26 | 24 | 12 | 16 | 21 | 22 | 20 |
| **9** | Surface antigen-like protein | E9AK01_LEIMU [2] | 74 | 36 | 26 | 2 | 39 | 29 | 26 | 19 | 39 | 19 |
| **10** | Glucose transporter, lmgt2 | E9AU63_LEIMU | 61 | 24 | 14 | 11 | 11 | 10 | 10 | 6 | 18 | 19 |
| **11** | Calpain-like cysteine peptidase | E9AUQ7_LEIMU [2] | 104 | 31 | 10 | 4 | 27 | 17 | 7 | 7 | 26 | 18 |
| **12** | Tubulin alpha chain | E9AP62_LEIMU | 60 | 19 | 10 | 13 | 25 | 18 | 17 | 16 | 21 | 17 |
| **13** | Actin | C6KJD5_LEIME (+2) | 42 | 42 | 11 | 2 | 29 | 18 | 23 | 14 | 41 | 16 |
| **14** | Putative calpain-like cysteine peptidase | E9AUS1_LEIMU | 15 | 11 | 10 | 4 | 13 | 9 | 9 | 9 | 9 | 16 |
| **15** | Elongation factor 2 | E9ASD6_LEIMU | 94 | 33 | 16 | 11 | 16 | 17 | 18 | 13 | 28 | 15 |
| **16** | Enolase | E9APW3_LEIMU | 46 | 18 | 11 | 2 | 16 | 15 | 8 | 5 | 23 | 15 |
| **17** | Tryparedoxin peroxidase | E9AQA6_LEIMU [2] | 22 | 18 | 10 | 8 | 12 | 10 | 8 | 7 | 12 | 13 |
| **18** | Putative ATPase alpha subunit | E8NHQ7_LEIMU (+1) | 63 | 12 | 9 | 17 | 9 | 7 | 8 | 11 | 5 | 13 |
| **19** | Putative nucleoside transporter 1 | E9ASW8_LEIMU | 54 | 8 | 17 | 15 | 8 | 10 | 10 | 11 | 10 | 13 |
| **20** | Surface antigen-like protein | E9AKM8_LEIMU | 30 | 21 | 15 | 2 | 20 | 17 | 12 | 17 | 17 | 11 |
| **21** | Peptidyl-prolyl cis-trans isomerase | E9AXG9_LEIMU | 19 | 12 | 8 | 4 | 7 | 5 | 3 | 2 | 10 | 11 |
| **22** | Putative histidine secretory acid phosphatase | E9AU82_LEIMU (+2) | 128 | 11 | 16 | 13 | 24 | 16 | 11 | 7 | 15 | 11 |
| **23** | Putative polyubiquitin | E9AMU1_LEIMU (+2) | 15 | 2 | 3 | 2 | 4 | 2 | 2 | 2 | 3 | 10 |
| **24** | Receptor-type adenylate cyclase a | E9ARD7_LEIMU [4] | 151 | 29 | 24 | 9 | 36 | 30 | 21 | 22 | 49 | 9 |
| **25** | Nucleoside diphosphate kinase | A0A1Y0DDB3_LEIAM (+1) | 17 | 3 | 5 | 1 | 4 | 2 | 4 | 2 | 5 | 9 |
| **26** | Putative cystathione gamma lyase | E9B6K2_LEIMU | 44 | 14 | 6 | 6 | 13 | 10 | 6 | 4 | 13 | 8 |
| **27** | Mitochondrial processing peptidase, beta subunit,putative,metallo-peptidase, Clan ME, Family M16 | E9B617_LEIMU | 55 | 7 | 3 | 7 | 3 | 2 | 3 | 5 | 2 | 7 |
| **28** | Glyceraldehyde-3-phosphate dehydrogenase | E9B170_LEIMU | 39 | 5 | 2 | 0 | 5 | 3 | 1 | 3 | 4 | 7 |
| **29** | Putative membrane-bound acid phosphatase 2 | E9AT36_LEIMU | 63 | 4 | 6 | 9 | 7 | 4 | 6 | 5 | 4 | 7 |
| **30** | Histone H4 | E9ASB9_LEIMU | 11 | 3 | 3 | 2 | 5 | 4 | 5 | 4 | 1 | 6 |
| **31** | Putative amino acid permease | E9AYW8_LEIMU [3] | 56 | 6 | 6 | 8 | 5 | 8 | 8 | 6 | 8 | 5 |
| **32** | Histone H2B | E9AR94_LEIMU [2] | 12 | 1 | 0 | 3 | 1 | 2 | 6 | 2 | 0 | 5 |
| **33** | WGS CADB00000000 data, contig 84 | E8NHQ5_LEIMU [2] | 76 | 1 | 5 | 9 | 3 | 4 | 2 | 2 | 0 | 5 |
| **34** | Putative long-chain-fatty-acid-CoA ligase | E9AJD9_LEIMU | 78 | 1 | 8 | 5 | 2 | 3 | 5 | 0 | 12 | 5 |
| **35** | Amastin-like surface protein-like protein | E9B0L6_LEIMU | 25 | 0 | 0 | 0 | 0 | 0 | 0 | 0 | 0 | 5 |
| **36** | I 6 autoantigen-like protein | E9AVX8_LEIMU | 23 | 0 | 1 | 0 | 0 | 0 | 0 | 0 | 4 | 5 |
| **37** | Putative calcium motive P-type ATPase | E9B686_LEIMU | 134 | 27 | 18 | 9 | 11 | 11 | 12 | 7 | 16 | 4 |
| **38** | Surface antigen-like protein | E9AKJ6_LEIMU | 31 | 15 | 7 | 1 | 22 | 16 | 11 | 17 | 20 | 4 |
| **39** | Putative casein kinase | E9B5Y1_LEIMU | 40 | 9 | 3 | 0 | 7 | 6 | 7 | 4 | 7 | 4 |
| **40** | Putative ADP,ATP carrier protein 1, mitochondrial | E9ARX1_LEIMU (+1) | 35 | 6 | 4 | 6 | 4 | 1 | 4 | 4 | 4 | 4 |
| **41** | Histone H2A | E9AV77_LEIMU [2] | 14 | 2 | 0 | 1 | 4 | 4 | 3 | 1 | 0 | 4 |
| **42** | Putative surface protein amastin | E9B0L4_LEIMU (+1) | 21 | 2 | 6 | 2 | 1 | 3 | 4 | 0 | 3 | 4 |
| **43** | Putative zinc transporter | E9B012_LEIMU | 47 | 0 | 0 | 0 | 0 | 0 | 0 | 0 | 0 | 4 |
| **44** | p-glycoprotein | E9B4S1_LEIMU [2] | 147 | 32 | 21 | 6 | 19 | 12 | 8 | 10 | 30 | 3 |
| **45** | Putative heat shock protein | E9ARS1_LEIMU | 92 | 8 | 10 | 3 | 15 | 7 | 5 | 4 | 12 | 3 |
| **46** | Ribonucleoprotein p18, mitochondrial,putative | E9AQ29_LEIMU | 21 | 8 | 2 | 5 | 0 | 0 | 3 | 4 | 2 | 3 |
| **47** | Superoxide dismutase | E9B2V9_LEIMU | 22 | 8 | 5 | 4 | 4 | 5 | 4 | 1 | 2 | 3 |
| **48** | Glycosomal phosphoenolpyruvate carboxykinase,putative | E9AZ81_LEIMU (+1) | 58 | 6 | 0 | 0 | 4 | 2 | 0 | 3 | 4 | 3 |
| **49** | Putative long-chain-fatty-acid-CoA ligase | E9AJD6_LEIMU | 77 | 6 | 3 | 0 | 2 | 3 | 1 | 0 | 4 | 3 |
| **50** | Adenosylhomocysteinase | E9ATH3_LEIMU | 48 | 5 | 6 | 3 | 7 | 4 | 4 | 4 | 5 | 3 |
| **51** | Clathrin heavy chain | E9AST4_LEIMU | 191 | 4 | 22 | 49 | 13 | 0 | 25 | 6 | 3 | 3 |
| **52** | Putative calpain-like cysteine peptidase | E9AUR8_LEIMU | 17 | 4 | 4 | 3 | 2 | 5 | 3 | 4 | 6 | 3 |
| **53** | Histone H4 | E9AKM9_LEIMU | 11 | 3 | 3 | 3 | 5 | 3 | 4 | 4 | 3 | 3 |
| **54** | Putative surface antigen (Fragment) | E9ANZ9_LEIMU [3] | 74 | 2 | 2 | 2 | 2 | 2 | 2 | 2 | 2 | 3 |
| **55** | Putative NADP-dependent alcohol dehydrogenase | E9AW40_LEIMU | 38 | 9 | 10 | 5 | 8 | 6 | 5 | 5 | 11 | 2 |
| **56** | Surface antigen-like protein | E9AK03_LEIMU | 37 | 9 | 2 | 2 | 7 | 7 | 2 | 2 | 14 | 2 |
| **57** | Putative kinesin K39 | E9APV6_LEIMU | 536 | 6 | 4 | 2 | 3 | 0 | 0 | 2 | 5 | 2 |
| **58** | WGS CADB00000000 data, contig 52 (Fragment) | E8NHK6_LEIMU | 209 | 6 | 5 | 3 | 6 | 2 | 3 | 5 | 6 | 2 |
| **59** | WGS CADB00000000 data, contig 72 (Fragment) | E8NHN7_LEIMU [2] | 96 | 5 | 3 | 2 | 6 | 5 | 3 | 6 | 5 | 2 |
| **60** | Putative small GTP-binding protein Rab1 | E9AYX8_LEIMU | 22 | 5 | 2 | 0 | 4 | 2 | 1 | 1 | 2 | 2 |
| **61** | Putative ADP-ribosylation factor-like (Putative small gtpase) | E9AQU9_LEIMU | 20 | 4 | 2 | 0 | 8 | 0 | 1 | 2 | 4 | 2 |
| **62** | Putative small GTP-binding protein Rab11 | E9ANA1_LEIMU | 23 | 3 | 2 | 2 | 6 | 0 | 2 | 1 | 4 | 2 |
| **63** | Amastin-like protein | E9B4S0_LEIMU | 21 | 2 | 2 | 2 | 2 | 1 | 2 | 0 | 2 | 2 |
| **64** | Putative proteophosphoglycan ppg3 (Fragment) | E9B5T5_LEIMU [2] | 120 | 1 | 2 | 0 | 6 | 2 | 2 | 3 | 6 | 2 |
| **65** | Protein tyrosine phosphatase-like protein | E9AQI0_LEIMU | 19 | 1 | 4 | 0 | 2 | 1 | 0 | 1 | 2 | 2 |
| **66** | Putative cytochrome c | E9AQU2_LEIMU | 12 | 1 | 2 | 2 | 1 | 1 | 1 | 1 | 0 | 2 |
| **67** | Putative rab1 small GTP-binding protein | E9ANC6_LEIMU | 25 | 1 | 3 | 0 | 1 | 0 | 1 | 2 | 0 | 2 |
| **68** | 3'-nucleotidase nuclease | Q9GNZ4_LEIME | 42 | 0 | 1 | 4 | 3 | 1 | 0 | 2 | 0 | 2 |
| **69** | Putative transmembrane amino acid transporter | E9ALD5_LEIMU | 54 | 0 | 1 | 0 | 0 | 1 | 0 | 0 | 0 | 2 |
| **70** | Universal minicircle sequence binding protein,putative | E9AST2_LEIMU | 13 | 0 | 0 | 0 | 0 | 1 | 0 | 0 | 2 | 2 |
| **71** | Putative myosin heavy chain | E9B3G5_LEIMU | 119 | 33 | 13 | 0 | 21 | 17 | 14 | 10 | 24 | 1 |
| **72** | Kinesin-like protein | E9AKI2_LEIMU | 154 | 19 | 6 | 2 | 19 | 12 | 9 | 9 | 21 | 1 |
| **73** | Putative ecotin | E9AQ49_LEIMU | 41 | 18 | 11 | 0 | 11 | 9 | 8 | 9 | 11 | 1 |
| **74** | Putative ADP ribosylation factor 3 | E9ALY6_LEIMU | 20 | 11 | 5 | 1 | 8 | 9 | 8 | 5 | 7 | 1 |
| **75** | Cluster of Proteasome regulatory ATPase subunit | E9ATM0_LEIMU [5] | 46 | 10 | 3 | 0 | 15 | 4 | 3 | 3 | 9 | 1 |
| **76** | Putative calpain-like cysteine peptidase (Putative cysteine peptidase, clan ca, family c2) | E9APT0_LEIMU | 13 | 9 | 2 | 1 | 6 | 2 | 4 | 4 | 7 | 1 |
| **77** | GDP-mannose pyrophosphorylase | Q9BLW4_LEIME | 41 | 8 | 4 | 0 | 3 | 3 | 2 | 1 | 3 | 1 |
| **78** | Putative calpain-like cysteine peptidase | E9AK26_LEIMU | 89 | 8 | 4 | 0 | 11 | 4 | 4 | 1 | 11 | 1 |
| **79** | Putative serine/threonine-protein kinase (Putative protein kinase) | E9AXW8_LEIMU | 44 | 7 | 4 | 3 | 9 | 7 | 2 | 1 | 6 | 1 |
| **80** | Putative ADP-ribosylation factor | E9B278_LEIMU | 20 | 6 | 3 | 4 | 5 | 2 | 2 | 3 | 5 | 1 |
| **81** | Putative aminopeptidase P | E9B6B5_LEIMU | 54 | 6 | 0 | 1 | 2 | 0 | 0 | 2 | 3 | 1 |
| **82** | Acetyl-coenzyme A synthetase | E9AW86_LEIMU | 77 | 5 | 3 | 0 | 5 | 0 | 0 | 0 | 2 | 1 |
| **83** | Putative glucose-regulated protein 78 | E9AZT9_LEIMU | 72 | 5 | 3 | 0 | 2 | 2 | 1 | 6 | 0 | 1 |
| **84** | Cluster of Phosphoglycerate kinase | E9AUF1_LEIMU [2] | 45 | 4 | 1 | 0 | 1 | 0 | 2 | 0 | 5 | 1 |
| **85** | S-adenosylmethionine synthase | E9B1C6_LEIMU | 43 | 4 | 5 | 3 | 3 | 1 | 2 | 2 | 4 | 1 |
| **86** | Phosphodiesterase | E9ALI0_LEIMU | 80 | 3 | 2 | 0 | 5 | 1 | 0 | 1 | 3 | 1 |
| **87** | Putative major vault protein | E9AKB0_LEIMU | 93 | 3 | 4 | 3 | 1 | 0 | 3 | 0 | 0 | 1 |
| **88** | Putative tyrosine aminotransferase | E9AT13_LEIMU | 50 | 3 | 4 | 1 | 7 | 6 | 5 | 3 | 3 | 1 |
| **89** | Mitochondrial processing peptidase alpha subunit,putative (Metallo-peptidase, clan me, family m16) | E9APB1_LEIMU | 58 | 2 | 1 | 0 | 2 | 0 | 0 | 3 | 0 | 1 |
| **90** | Proteasome endopeptidase complex | E9AYS1_LEIMU | 26 | 2 | 0 | 0 | 4 | 0 | 0 | 0 | 3 | 1 |
| **91** | Putative adenylate kinase | E9AVA9_LEIMU | 30 | 2 | 0 | 0 | 0 | 1 | 1 | 1 | 2 | 1 |
| **92** | Putative ATP-binding cassette protein subfamily G, member 2 | E9AKN7_LEIMU | 74 | 2 | 0 | 0 | 2 | 0 | 0 | 0 | 0 | 1 |
| **93** | Putative c2 domain protein | E9B1N1_LEIMU | 30 | 2 | 2 | 2 | 2 | 1 | 0 | 2 | 2 | 1 |
| **94** | Phospholipid-transporting ATPase | E9APH7_LEIMU | 125 | 1 | 0 | 1 | 1 | 0 | 0 | 0 | 0 | 1 |
| **95** | Putative cysteine synthase | E9ATD8_LEIMU | 35 | 1 | 0 | 0 | 2 | 0 | 0 | 0 | 1 | 1 |
| **96** | Putative isoleucyl-tRNA synthetase | E9ATZ6_LEIMU | 126 | 1 | 2 | 2 | 1 | 0 | 2 | 0 | 2 | 1 |
| **97** | Autophagy-related protein | E9AS34_LEIMU (+2) | 12 | 0 | 1 | 0 | 2 | 1 | 0 | 1 | 3 | 1 |
| **98** | GTP-binding nuclear protein | E9AXM1_LEIMU | 24 | 0 | 0 | 0 | 0 | 0 | 0 | 0 | 0 | 1 |
| **99** | Putative 3'-nucleotidase/nuclease | E9ANW7_LEIMU | 41 | 0 | 3 | 1 | 4 | 1 | 2 | 1 | 2 | 1 |
| **100** | Putative 40S ribosomal protein L14 | E9AVY4_LEIMU | 20 | 0 | 0 | 0 | 3 | 2 | 3 | 0 | 0 | 1 |
| **101** | Putative 40S ribosomal protein S4 | E9APE6_LEIMU | 47 | 0 | 0 | 3 | 10 | 5 | 4 | 2 | 0 | 1 |
| **102** | Putative 60S ribosomal protein L21 | E9AQK4_LEIMU | 18 | 0 | 2 | 3 | 2 | 4 | 4 | 2 | 0 | 1 |
| **103** | Transmembrane 9 superfamily member | E9ALR2_LEIMU | 77 | 0 | 0 | 0 | 0 | 0 | 0 | 0 | 0 | 1 |
| **104** | Cluster of 2,3-bisphosphoglycerate-independent phosphoglycerate mutase | E9AUA1_LEIMU [2] | 61 | 16 | 6 | 1 | 10 | 4 | 2 | 4 | 9 | 0 |
| **105** | Transketolase | E9AX52_LEIMU (+1) | 72 | 16 | 4 | 0 | 10 | 3 | 1 | 2 | 6 | 0 |
| **106** | Heat shock protein 70-related protein | E9AYA3_LEIMU | 71 | 15 | 6 | 1 | 10 | 7 | 7 | 3 | 10 | 0 |
| **107** | Cluster of Pyruvate kinase | E9B5P1_LEIMU [2] | 54 | 12 | 7 | 4 | 9 | 8 | 5 | 5 | 6 | 0 |
| **108** | Putative ABC transporter | E9AYZ8_LEIMU | 203 | 12 | 7 | 6 | 16 | 13 | 8 | 7 | 13 | 0 |
| **109** | Putative seryl-tRNA synthetase | E9ANF6_LEIMU | 53 | 12 | 6 | 2 | 11 | 4 | 4 | 6 | 11 | 0 |
| **110** | Transitional endoplasmic reticulum ATPase,putative | E9ASQ6_LEIMU | 87 | 11 | 3 | 0 | 9 | 3 | 0 | 0 | 5 | 0 |
| **111** | Cluster of 6-phosphogluconate dehydrogenase, decarboxylating | E9B6L3_LEIMU [3] | 52 | 10 | 2 | 0 | 4 | 1 | 0 | 2 | 4 | 0 |
| **112** | Putative casein kinase I | E9AXN8_LEIMU | 59 | 10 | 6 | 0 | 10 | 4 | 1 | 3 | 5 | 0 |
| **113** | Phosphotransferase | E9AUZ2_LEIMU | 52 | 8 | 0 | 0 | 4 | 0 | 0 | 0 | 4 | 0 |
| **114** | Putative calpain-like cysteine peptidase | E9AUQ9_LEIMU | 78 | 8 | 5 | 0 | 12 | 11 | 9 | 6 | 13 | 0 |
| **115** | Putative ecotin | E9AQ32_LEIMU | 16 | 8 | 3 | 0 | 4 | 0 | 0 | 1 | 1 | 0 |
| **116** | Cluster of Putative ABC transporter | E9ANR6_LEIMU [2] | 211 | 7 | 2 | 0 | 12 | 4 | 3 | 0 | 5 | 0 |
| **117** | Serine/threonine-protein phosphatase | E9AKB4_LEIMU | 72 | 7 | 4 | 3 | 7 | 4 | 2 | 5 | 5 | 0 |
| **118** | Malic enzyme | E9AWR7_LEIMU | 63 | 6 | 1 | 0 | 5 | 1 | 1 | 0 | 1 | 0 |
| **119** | Phosphodiesterase | E9ARP6_LEIMU | 71 | 6 | 7 | 0 | 8 | 7 | 4 | 6 | 9 | 0 |
| **120** | Proteasome regulatory non-ATPase subunit 6,putative | E9AJK0_LEIMU | 59 | 6 | 3 | 0 | 2 | 2 | 3 | 2 | 2 | 0 |
| **121** | Putative ABC transporter | E9ANR4_LEIMU | 200 | 6 | 0 | 0 | 7 | 2 | 0 | 1 | 6 | 0 |
| **122** | Putative thimet oligopeptidase (Metallo-peptidase, clan ma(E), family m3) | E9AYD6_LEIMU | 77 | 6 | 2 | 0 | 8 | 1 | 0 | 0 | 2 | 0 |
| **123** | Transmembrane 9 superfamily member | E9B5F7_LEIMU | 71 | 6 | 2 | 1 | 0 | 0 | 0 | 0 | 2 | 0 |
| **124** | Dipeptidyl peptidase 3 | E9AKK2_LEIMU | 76 | 5 | 3 | 0 | 5 | 0 | 3 | 2 | 4 | 0 |
| **125** | Proteasome regulatory non-ATP-ase subunit 2,putative | E9AZZ2_LEIMU | 108 | 5 | 0 | 0 | 6 | 1 | 0 | 0 | 3 | 0 |
| **126** | Proteasome subunit alpha type | E9AVH9_LEIMU | 27 | 5 | 3 | 2 | 8 | 2 | 2 | 2 | 8 | 0 |
| **127** | Proteasome subunit beta | E9B5M9_LEIMU | 25 | 5 | 4 | 2 | 3 | 2 | 2 | 0 | 2 | 0 |
| **128** | Putative aminopeptidase (Metallo-peptidase, clan mf, family m17) | E9ANK8_LEIMU | 57 | 5 | 3 | 2 | 0 | 0 | 1 | 0 | 2 | 0 |
| **129** | Putative dual specificity protein phosphatase | E9B505_LEIMU | 153 | 5 | 0 | 0 | 5 | 0 | 0 | 0 | 5 | 0 |
| **130** | Rab GDP dissociation inhibitor | E9ALK7_LEIMU | 50 | 5 | 0 | 0 | 4 | 0 | 0 | 0 | 6 | 0 |
| **131** | Serine/threonine-protein phosphatase | E9ANZ3_LEIMU | 109 | 5 | 5 | 1 | 2 | 0 | 2 | 0 | 5 | 0 |
| **132** | Transaldolase | E9AQN5_LEIMU | 37 | 5 | 1 | 0 | 7 | 1 | 2 | 2 | 3 | 0 |
| **133** | Trypanothione reductase | E9AKE1_LEIMU | 53 | 5 | 4 | 2 | 6 | 3 | 1 | 2 | 5 | 0 |
| **134** | Tryparedoxin | E9ALV7_LEIMU | 17 | 5 | 2 | 0 | 2 | 2 | 1 | 0 | 1 | 0 |
| **135** | Aminopeptidase | E9ALJ9_LEIMU | 96 | 4 | 2 | 0 | 2 | 2 | 1 | 0 | 4 | 0 |
| **136** | Biotin/lipoate protein ligase-like protein | E9B1Q4_LEIMU | 28 | 4 | 1 | 0 | 2 | 0 | 0 | 0 | 1 | 0 |
| **137** | Cluster of Putative NADH-dependent fumarate reductase | E9B5Z7_LEIMU [2] | 123 | 4 | 0 | 0 | 2 | 0 | 0 | 0 | 4 | 0 |
| **138** | D-lactate dehydrogenase-like protein | E9AZA6_LEIMU | 53 | 4 | 1 | 0 | 2 | 0 | 1 | 1 | 0 | 0 |
| **139** | Dihydroorotate dehydrogenase (fumarate) | E9AQL1_LEIMU | 34 | 4 | 0 | 0 | 2 | 0 | 0 | 0 | 1 | 0 |
| **140** | Palmitoyltransferase | E9AWG3_LEIMU | 49 | 4 | 0 | 0 | 0 | 0 | 0 | 0 | 1 | 0 |
| **141** | Proteasome regulatory non-ATP-ase subunit 5,putative | E9AV44_LEIMU | 54 | 4 | 5 | 0 | 9 | 1 | 0 | 0 | 6 | 0 |
| **142** | Proteasome subunit alpha type | E9AVG6_LEIMU | 25 | 4 | 2 | 2 | 6 | 3 | 2 | 0 | 7 | 0 |
| **143** | Proteasome subunit alpha type | E9AST1_LEIMU | 30 | 4 | 0 | 0 | 1 | 0 | 0 | 0 | 2 | 0 |
| **144** | Putative 5-methyltetrahydropteroyltriglutamate--homocyst ei nemethyltransferase | E9B1E9_LEIMU | 86 | 4 | 0 | 0 | 5 | 2 | 1 | 1 | 3 | 0 |
| **145** | Putative adenosine kinase | E9B0L7_LEIMU | 37 | 4 | 4 | 6 | 5 | 5 | 3 | 4 | 5 | 0 |
| **146** | Putative ATP-dependent Clp protease subunit, heat shock protein 100 (HSP100) | E9ALU6_LEIMU | 97 | 4 | 0 | 0 | 0 | 0 | 0 | 0 | 0 | 0 |
| **147** | Putative dynein heavy chain | E9API9_LEIMU | 530 | 4 | 0 | 0 | 4 | 2 | 0 | 1 | 4 | 0 |
| **148** | Putative dynein heavy chain | E9AXH6_LEIMU | 537 | 4 | 0 | 0 | 12 | 0 | 0 | 0 | 4 | 0 |
| **149** | Putative ef-hand protein 5 | E9AMU2_LEIMU | 21 | 4 | 4 | 0 | 4 | 1 | 1 | 0 | 5 | 0 |
| **150** | Putative Gim5A protein (Glycosomal membrane protein) | E9B6P8_LEIMU | 25 | 4 | 0 | 0 | 3 | 0 | 0 | 0 | 0 | 0 |
| **151** | Putative GTP-binding protein | E9ALK3_LEIMU | 77 | 4 | 2 | 2 | 2 | 0 | 4 | 2 | 0 | 0 |
| **152** | Putative sarcoplasmic reticulum glycoprotein | E9ARX9_LEIMU | 69 | 4 | 1 | 0 | 4 | 1 | 0 | 0 | 0 | 0 |
| **153** | Putative serine/threonine-protein kinase | E9B296_LEIMU | 50 | 4 | 1 | 0 | 5 | 1 | 1 | 1 | 5 | 0 |
| **154** | Surface antigen-like protein | E9AK02_LEIMU | 18 | 4 | 3 | 0 | 2 | 2 | 2 | 4 | 4 | 0 |
| **155** | 6-phosphogluconolactonase | E9AYQ1_LEIMU | 28 | 3 | 0 | 0 | 2 | 0 | 0 | 0 | 0 | 0 |
| **156** | ADP-ribosylation factor-like protein | E9B110_LEIMU | 20 | 3 | 1 | 0 | 3 | 0 | 0 | 0 | 1 | 0 |
| **157** | Alanine--tRNA ligase | E9AVY6_LEIMU | 106 | 3 | 1 | 0 | 3 | 0 | 1 | 1 | 4 | 0 |
| **158** | ATP-dependent 6-phosphofructokinase | E9ALH3_LEIMU | 54 | 3 | 3 | 2 | 1 | 0 | 1 | 0 | 1 | 0 |
| **159** | Miltefosine transporter beta subunit | A0A075DN10_LEIAM (+1) | 40 | 3 | 3 | 2 | 2 | 0 | 2 | 2 | 2 | 0 |
| **160** | Obg-like ATPase 1 | E9AZE0_LEIMU | 44 | 3 | 0 | 0 | 4 | 2 | 0 | 0 | 1 | 0 |
| **161** | Putative 60S ribosomal protein L13a | E9AQ19_LEIMU | 26 | 3 | 2 | 0 | 1 | 0 | 2 | 0 | 3 | 0 |
| **162** | Putative adenylate kinase | E9B4H8_LEIMU | 25 | 3 | 2 | 1 | 4 | 0 | 2 | 4 | 2 | 0 |
| **163** | Putative aminoacylase | E9AUV8_LEIMU | 43 | 3 | 0 | 0 | 1 | 0 | 0 | 0 | 3 | 0 |
| **164** | Putative arginyl-tRNA synthetase | E9AZ37_LEIMU | 78 | 3 | 0 | 0 | 2 | 0 | 0 | 0 | 0 | 0 |
| **165** | Putative paraflagellar rod protein | E9AKJ8_LEIMU | 117 | 3 | 2 | 0 | 10 | 2 | 1 | 1 | 6 | 0 |
| **166** | Putative peptidyl dipeptidase | E9AJN6_LEIMU | 77 | 3 | 0 | 0 | 3 | 1 | 1 | 0 | 2 | 0 |
| **167** | Putative peptidyl-prolyl cis-trans isomerase (Cyclophilin-40) | E9B705_LEIMU | 39 | 3 | 4 | 1 | 3 | 0 | 2 | 0 | 2 | 0 |
| **168** | Putative proteasome regulatory ATPase subunit 2 | E9APD3_LEIMU | 49 | 3 | 0 | 0 | 2 | 0 | 0 | 0 | 4 | 0 |
| **169** | Putative Qc-SNARE protein | E9AM81_LEIMU | 24 | 3 | 2 | 3 | 2 | 1 | 4 | 2 | 2 | 0 |
| **170** | Putative trypanothione synthetase | E9AZ89_LEIMU | 74 | 3 | 2 | 1 | 1 | 2 | 0 | 0 | 4 | 0 |
| **171** | Putative tyrosyl-tRNA synthetase | E9APY5_LEIMU | 75 | 3 | 0 | 0 | 2 | 0 | 0 | 0 | 1 | 0 |
| **172** | Stress-inducible protein STI1 homolog | E9ASC5_LEIMU | 29 | 3 | 2 | 0 | 2 | 0 | 0 | 0 | 2 | 0 |
| **173** | 14-3-3 protein-like protein | E9AT99_LEIMU | 30 | 2 | 0 | 0 | 1 | 0 | 0 | 0 | 2 | 0 |
| **174** | Aconitate hydratase | E9ARI8_LEIMU | 97 | 2 | 0 | 0 | 0 | 0 | 0 | 0 | 2 | 0 |
| **175** | Autophagy-related protein | E9AML6_LEIMU (+1) | 13 | 2 | 2 | 0 | 2 | 2 | 2 | 2 | 2 | 0 |
| **176** | Autophagy-related protein | E9AS40_LEIMU | 15 | 2 | 2 | 0 | 3 | 1 | 3 | 1 | 4 | 0 |
| **177** | D-3-phosphoglycerate dehydrogenase-like protein | E9AJN9_LEIMU | 44 | 2 | 2 | 0 | 3 | 0 | 2 | 1 | 2 | 0 |
| **178** | Fructose-bisphosphate aldolase | E9ASP6_LEIMU | 41 | 2 | 0 | 0 | 0 | 0 | 0 | 0 | 0 | 0 |
| **179** | Glyceraldehyde-3-phosphate dehydrogenase-like protein | E9B703_LEIMU | 36 | 2 | 0 | 0 | 0 | 0 | 0 | 0 | 1 | 0 |
| **180** | NADH:flavin oxidoreductase/NADH oxidase,putative | E9AP13_LEIMU | 41 | 2 | 0 | 0 | 2 | 0 | 1 | 0 | 2 | 0 |
| **181** | Phosphatidylinositol-4-phosphate 5-kinase-like protein | E9ASF4_LEIMU | 51 | 2 | 0 | 0 | 0 | 1 | 0 | 0 | 1 | 0 |
| **182** | Phosphomannomutase | E9ASX1_LEIMU | 28 | 2 | 2 | 0 | 1 | 0 | 0 | 2 | 1 | 0 |
| **183** | Proteasome regulatory non-ATP-ase subunit 11,putative (19s proteasome regulatory subunit,metallo-peptidase, clan mp, family m67) | E9B4N4_LEIMU | 35 | 2 | 0 | 0 | 4 | 0 | 0 | 0 | 0 | 0 |
| **184** | Proteasome subunit beta type | E9AKP2_LEIMU | 28 | 2 | 0 | 0 | 2 | 0 | 0 | 0 | 0 | 0 |
| **185** | Proteasome subunit beta type | E9ANS8_LEIMU | 31 | 2 | 0 | 0 | 3 | 0 | 1 | 0 | 1 | 0 |
| **186** | Proteasome subunit beta type | E9AST7_LEIMU | 34 | 2 | 2 | 0 | 0 | 0 | 0 | 0 | 0 | 0 |
| **187** | Protein disulfide-isomerase | E9AUD1_LEIMU | 52 | 2 | 0 | 0 | 0 | 0 | 0 | 0 | 0 | 0 |
| **188** | Putative aspartyl-tRNA synthetase | E9B0H1_LEIMU | 62 | 2 | 0 | 0 | 1 | 0 | 0 | 0 | 0 | 0 |
| **189** | Putative cAMP-specific phosphodiesterase | E9AJY5_LEIMU | 73 | 2 | 0 | 0 | 2 | 0 | 0 | 0 | 2 | 0 |
| **190** | Putative carboxypeptidase | E9B493_LEIMU | 57 | 2 | 0 | 0 | 0 | 0 | 0 | 0 | 0 | 0 |
| **191** | Putative endoribonuclease L-PSP (Pb5) | E9AW21_LEIMU | 17 | 2 | 0 | 2 | 2 | 1 | 2 | 0 | 2 | 0 |
| **192** | Putative fatty acyl CoA syntetase 1 | E9AJD4_LEIMU | 77 | 2 | 2 | 0 | 5 | 2 | 2 | 1 | 1 | 0 |
| **193** | Putative heat shock protein DNAJ | E9AZE9_LEIMU | 44 | 2 | 2 | 0 | 5 | 4 | 1 | 1 | 3 | 0 |
| **194** | Putative lipophosphoglycan biosynthetic protein | E9AM02_LEIMU | 87 | 2 | 0 | 0 | 0 | 0 | 0 | 0 | 0 | 0 |
| **195** | Putative short chain dehydrogenase | E9B601_LEIMU | 25 | 2 | 0 | 0 | 0 | 0 | 0 | 0 | 0 | 0 |
| **196** | Putative ubiquitin-activating enzyme e1 | E9B6I5_LEIMU | 127 | 2 | 1 | 0 | 2 | 0 | 0 | 0 | 0 | 0 |
| **197** | Putative ubiquitin-conjugating enzyme e2 | E9B609_LEIMU | 17 | 2 | 2 | 0 | 2 | 2 | 2 | 0 | 1 | 0 |
| **198** | Reticulon-like protein | E9B128_LEIMU | 22 | 2 | 2 | 0 | 2 | 1 | 1 | 0 | 2 | 0 |
| **199** | Serine hydroxymethyltransferase | E9APY0_LEIMU | 51 | 2 | 1 | 2 | 1 | 1 | 0 | 0 | 0 | 0 |
| **200** | 40S ribosomal protein S2 | E9ARV7_LEIMU (+2) | 33 | 1 | 0 | 0 | 2 | 0 | 1 | 1 | 0 | 0 |
| **201** | Cluster of Putative surface antigen protein 2 | E9AP03_LEIMU [4] | 41 | 1 | 0 | 2 | 0 | 1 | 0 | 2 | 0 | 0 |
| **202** | Glutathione peroxidase | E9AY59_LEIMU (+1) | 20 | 1 | 0 | 0 | 0 | 0 | 0 | 2 | 2 | 0 |
| **203** | Kinesin-like protein | E9ARX7_LEIMU | 94 | 1 | 1 | 2 | 1 | 1 | 3 | 2 | 4 | 0 |
| **204** | Peptidylprolyl isomerase | E9ASA6_LEIMU | 48 | 1 | 1 | 0 | 2 | 0 | 0 | 0 | 2 | 0 |
| **205** | Phosphodiesterase | E9AQE4_LEIMU | 103 | 1 | 0 | 0 | 2 | 1 | 0 | 0 | 0 | 0 |
| **206** | Proteasome regulatory non-ATPase subunit,putative | E9AM67_LEIMU | 99 | 1 | 0 | 0 | 4 | 2 | 0 | 0 | 1 | 0 |
| **207** | Putative 40S ribosomal protein S15A | E9ALP4_LEIMU | 15 | 1 | 0 | 1 | 1 | 0 | 1 | 3 | 1 | 0 |
| **208** | Putative glycogen synthase kinase 3 beta | Q0PKV3_LEIME | 41 | 1 | 0 | 0 | 2 | 0 | 0 | 0 | 1 | 0 |
| **209** | Putative glycyl tRNA synthetase | E9ATG6_LEIMU | 70 | 1 | 1 | 0 | 2 | 0 | 1 | 0 | 0 | 0 |
| **210** | Putative long chain fatty Acyl CoA synthetase | E9AJQ8_LEIMU | 74 | 1 | 2 | 0 | 2 | 2 | 2 | 1 | 4 | 0 |
| **211** | Putative nucleoside transporter 1 | E9AQB6_LEIMU | 54 | 1 | 2 | 4 | 0 | 3 | 2 | 2 | 0 | 0 |
| **212** | Putative oxidoreductase | E9ATK1_LEIMU | 36 | 1 | 1 | 2 | 2 | 0 | 0 | 0 | 1 | 0 |
| **213** | Putative paraflagellar rod protein 1D | E9ALP7_LEIMU (+1) | 69 | 1 | 0 | 0 | 3 | 1 | 0 | 0 | 3 | 0 |
| **214** | Putative peptidase M20/M25/M40 | E9B3Z8_LEIMU | 52 | 1 | 0 | 0 | 1 | 0 | 0 | 0 | 3 | 0 |
| **215** | Putative phosphatase 2C | E9B0G2_LEIMU | 42 | 1 | 0 | 0 | 2 | 0 | 0 | 0 | 1 | 0 |
| **216** | Putative proteasome regulatory ATPase subunit 5 | E9AVP4_LEIMU | 49 | 1 | 1 | 0 | 2 | 2 | 0 | 1 | 0 | 0 |
| **217** | Putative serine/threonine protein kinase | E9B2K5_LEIMU | 149 | 1 | 0 | 0 | 6 | 0 | 0 | 0 | 0 | 0 |
| **218** | Putative serine/threonine-protein kinase | E9ALG7_LEIMU | 56 | 1 | 0 | 0 | 2 | 0 | 0 | 0 | 0 | 0 |
| **219** | Putative small GTP-binding protein Rab28 | E9B0U3_LEIMU | 26 | 1 | 0 | 0 | 3 | 2 | 1 | 2 | 4 | 0 |
| **220** | Ubiquitinyl hydrolase 1 | E9ALJ4_LEIMU | 81 | 1 | 0 | 0 | 3 | 0 | 0 | 0 | 1 | 0 |
| **221** | WGS CADB00000000 data, contig 73 | E8NHP1_LEIMU (+1) | 94 | 1 | 0 | 0 | 2 | 0 | 0 | 0 | 0 | 0 |
| **222** | 40S ribosomal protein S24 | E9AT64_LEIMU | 16 | 0 | 0 | 0 | 3 | 3 | 1 | 2 | 0 | 0 |
| **223** | 40S ribosomal protein S6 | E9AVH4_LEIMU | 28 | 0 | 0 | 3 | 3 | 1 | 2 | 2 | 0 | 0 |
| **224** | 40S ribosomal protein S8 | E9AX53_LEIMU | 25 | 0 | 0 | 0 | 4 | 1 | 2 | 3 | 0 | 0 |
| **225** | 60S ribosomal protein L11 (L5, L16) | E9AK29_LEIMU | 22 | 0 | 0 | 1 | 0 | 1 | 1 | 2 | 0 | 0 |
| **226** | Adenine phosphoribosyltransferase | E9AXZ5_LEIMU | 26 | 0 | 0 | 1 | 0 | 0 | 2 | 0 | 0 | 0 |
| **227** | Cluster of Kinesin-like protein | E9AR52_LEIMU [2] | 105 | 0 | 0 | 0 | 3 | 0 | 0 | 0 | 0 | 0 |
| **228** | Cofilin-like protein | E9AM28_LEIMU | 16 | 0 | 0 | 0 | 0 | 2 | 0 | 0 | 2 | 0 |
| **229** | Conserved TPR domain protein | E9B146_LEIMU | 45 | 0 | 0 | 0 | 3 | 0 | 0 | 0 | 0 | 0 |
| **230** | Eukaryotic translation initiation factor 5A | IF5A_LEIMU | 18 | 0 | 0 | 1 | 1 | 2 | 2 | 0 | 0 | 0 |
| **231** | p-glycoprotein-like protein (Abc transporter-like protein) (Multidrug resistance protein-like protein) | E9AYP8_LEIMU | 140 | 0 | 0 | 0 | 4 | 1 | 0 | 0 | 1 | 0 |
| **232** | Phosphate transporter | E9ANE4_LEIMU | 52 | 0 | 3 | 3 | 0 | 0 | 0 | 0 | 0 | 0 |
| **233** | Proteasome subunit alpha type | E9APM4_LEIMU | 32 | 0 | 1 | 0 | 0 | 0 | 1 | 0 | 2 | 0 |
| **234** | Putative 40S ribosomal protein S3 | E9AQ94_LEIMU | 24 | 0 | 0 | 0 | 2 | 0 | 0 | 1 | 0 | 0 |
| **235** | Putative 60S ribosomal protein L10 | E9AK58_LEIMU | 25 | 0 | 0 | 0 | 4 | 0 | 1 | 0 | 0 | 0 |
| **236** | Putative 60S ribosomal protein L13 | E9ALH7_LEIMU | 25 | 0 | 0 | 2 | 6 | 3 | 1 | 2 | 0 | 0 |
| **237** | Putative 60S ribosomal protein L19 | E9AKR8_LEIMU | 28 | 0 | 0 | 0 | 2 | 1 | 0 | 0 | 0 | 0 |
| **238** | Putative adenylate kinase | E9AK77_LEIMU | 23 | 0 | 0 | 0 | 1 | 0 | 0 | 0 | 2 | 0 |
| **239** | Putative asparagine synthetase a (Putative aspartate--ammonia ligase) | E9AY61_LEIMU | 40 | 0 | 0 | 0 | 0 | 0 | 0 | 0 | 2 | 0 |
| **240** | Putative ATP-dependent DEAD-box RNA helicase | E9B5S5_LEIMU | 46 | 0 | 0 | 0 | 2 | 0 | 0 | 0 | 1 | 0 |
| **241** | Putative calpain-like cysteine peptidase (Fragment) | E9AYV1_LEIMU | 269 | 0 | 0 | 0 | 1 | 0 | 0 | 0 | 2 | 0 |
| **242** | Putative calpain-like cysteine peptidase | E9B1J0_LEIMU | 89 | 0 | 0 | 0 | 3 | 1 | 0 | 0 | 1 | 0 |
| **243** | Putative glucosamine-fructose-6-phosphate aminotransferase | E9AKX3_LEIMU | 73 | 0 | 0 | 0 | 3 | 0 | 0 | 0 | 2 | 0 |
| **244** | Putative mitotubule-associated protein Gb4 | E9AYH5_LEIMU | 321 | 0 | 0 | 0 | 1 | 1 | 0 | 0 | 0 | 0 |
| **245** | Putative Pyridoxal kinase | E9B0Q4_LEIMU | 33 | 0 | 0 | 1 | 1 | 0 | 0 | 0 | 2 | 0 |
| **246** | Putative ribosomal protein L3 | E9B396_LEIMU | 48 | 0 | 0 | 0 | 2 | 0 | 1 | 0 | 0 | 0 |
| **247** | Putative serine/threonine-protein kinase | E9AVE6_LEIMU | 90 | 0 | 0 | 0 | 0 | 0 | 0 | 0 | 2 | 0 |
| **248** | Receptor-type adenylate cyclase a-like protein | E9AT96_LEIMU | 152 | 0 | 1 | 0 | 1 | 0 | 2 | 1 | 2 | 0 |
| **249** | S-methyl-5'-thioadenosine phosphorylase | E9AKI9_LEIMU | 33 | 0 | 0 | 2 | 0 | 0 | 0 | 0 | 0 | 0 |
| **250** | Succinyl-diaminopimelate desuccinylase-like protein | E9B201_LEIMU | 51 | 0 | 2 | 0 | 2 | 0 | 0 | 0 | 2 | 0 |
| **251** | Sucrose-phosphate synthase-like protein | A0A0R6XPE8_LEIME | 52 | 0 | 0 | 0 | 2 | 0 | 0 | 0 | 2 | 0 |
| **252** | WGS CADB00000000 data, contig 24 (Fragment) | E8NHG9_LEIMU | 80 | 0 | 0 | 0 | 3 | 0 | 0 | 0 | 0 | 0 |
| **253** | WGS CADB00000000 data, contig 99 | E8NHS7_LEIMU (+1) | 63 | 0 | 0 | 2 | 0 | 0 | 1 | 0 | 0 | 0 |

*Supplemental Table 1. All proteins identified from* L. Mexicana *Clones and Ht EVs represented using total spectrum count. Each protein was identified with 95% confidence and at least 2 detected peptides. Accession numbers are given in Uniprot format.*
